# Application of novel silica stabilized on a covalent triazine framework as a highly efficient heterogeneous and recyclable catalyst in the effective green synthesis of porphyrins†

**DOI:** 10.1039/d4ra07875f

**Published:** 2025-01-13

**Authors:** Azin Kharazmi, Ramin Ghorbani-Vaghei, Ardeshir Khazaei, Idris Karakaya, Rahman Karimi-Nami

**Affiliations:** a Department of Organic Chemistry, Faculty of Chemistry and Petroleum Sciences, Bu-Ali Sina University Hamedan 6517838683 Iran rgvaghei@yahoo.com ghorbani@basu.ac.ir +988138380709 +989183122123; b Department of Organic Chemistry, Faculty of Chemistry, University of Guilan Rasht Iran; c Department of Chemistry, College of Basic Sciences, Gebze Technical University 41400 Gebze Turkey; d Department of Chemistry, Faculty of Science, University of Maragheh P.O Box 55181-83111 Maragheh Iran

## Abstract

In this study, we present the design, synthesis, and utilization of a covalent triazine framework (CTF) formed by the condensation of *N*^2^,*N*^4^,*N*^6^-tris(4-(aminomethyl)benzyl)-1,3,5-triazine-2,4,6-triamine and 2,4,6-tris(4-formylphenoxy)-1,3,5-triazine on which silica is immobilized (TPT-TAT/silica) as an innovative catalyst for porphyrins synthesis. Under solvothermal conditions, the condensation of triamine and trialdehyde precursors led to the formation of a covalent triazine framework (CTF) with a high nitrogen content. The resulting CTF is characterized by its extensive porosity and elevated nitrogen levels, which are critical for the creation of catalytic active sites. This framework demonstrated exceptional catalytic performance in the synthesis of porphyrins. Substituting aerobic conditions *in lieu* of costly oxidizing agents represents a significant advancement in our methodology. Due to the insolubility of the catalyst, it is possible to separate it from the reaction mixture through filtration or centrifugation. This property enhances its reusability and minimizes waste generation. This development in the synthesis and application of CTFs could pave the way for more sustainable and cost-effective catalytic processes in organic synthesis, particularly in the synthesis of complex molecules like porphyrins. The research highlights the potential of CTFs as versatile materials in catalysis, owing to their structural properties and the ability to tailor their functionalities for specific applications.

## Abstract 2

In this study, we present the design, synthesis, and utilization of a covalent triazine framework (CTF) as an innovative catalyst for porphyrins synthesis.

## Graphical abstract

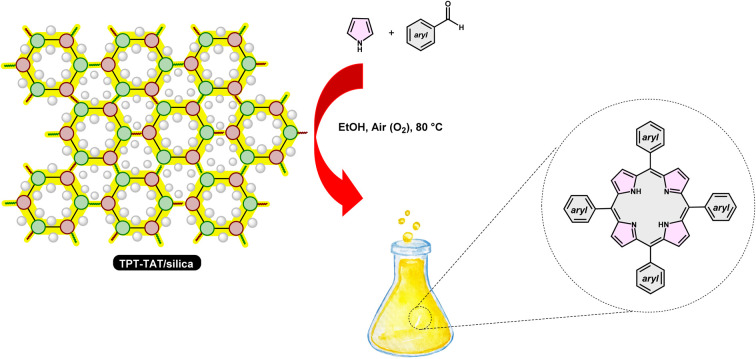

## Introduction

In recent decades, a new class of nanoporous materials known as porous organic polymers (POPs) has emerged. Their extensive porosity and covalent bonding confer a high surface area and exceptional stability, rendering them highly attractive for current research and applications.^1,2^ POP_S_ are divided into several subgroups, including conjugated microporous polymers (CMPs),^3^ porous aromatic frameworks (PAF_S_),^4^ internal microporous polymers (PIMS),^5^ crosslinked polymers (HCPS),^6^ covalent organic frameworks (COF_S_)^7,8^ and covalent triazine frameworks (CTF_S_).^9^ Among these subgroups, covalent triazine frameworks (CTFs) are distinguished by their unique porous structure, attributed to the 1,3,5-triazine units with π-conjugation properties, which enhance their chemical stability.^10–13^ Extensive literature demonstrates that covalent triazine frameworks (CTFs) are utilized in a variety of advanced applications, including energy storage and conversion,^14–17^ gas storage,^18–21^ photocatalysis,^22–26^ heterogeneous catalysis,^27–29^ sensors,^30^ optoelectronics.^31–34^ In addition, their rich nitrogen content, high surface area and high porosity help to stabilize active species.

Since the early 1960s, molten salts have been used to form triazines from nitrile compounds.^35^ In 1973, G. H. Miller established metal chlorides as effective catalysts for the trimerization of aromatic nitriles,^36^ and later, in 2008, Thomas and colleagues expanded on this by synthesizing covalent triazine frameworks (CTFs) from terephthalonitrile *via* thermal ion polymerization, articulating the concept of CTFs for the first time.^37^ A key advantage of covalent triazine frameworks (CTFs) is their modular design, which allows for the customization of building blocks and synthetic pathways; consequently, various methods have been developed to synthesize stable CTFs with diverse topologies.

**Fig. 1:**
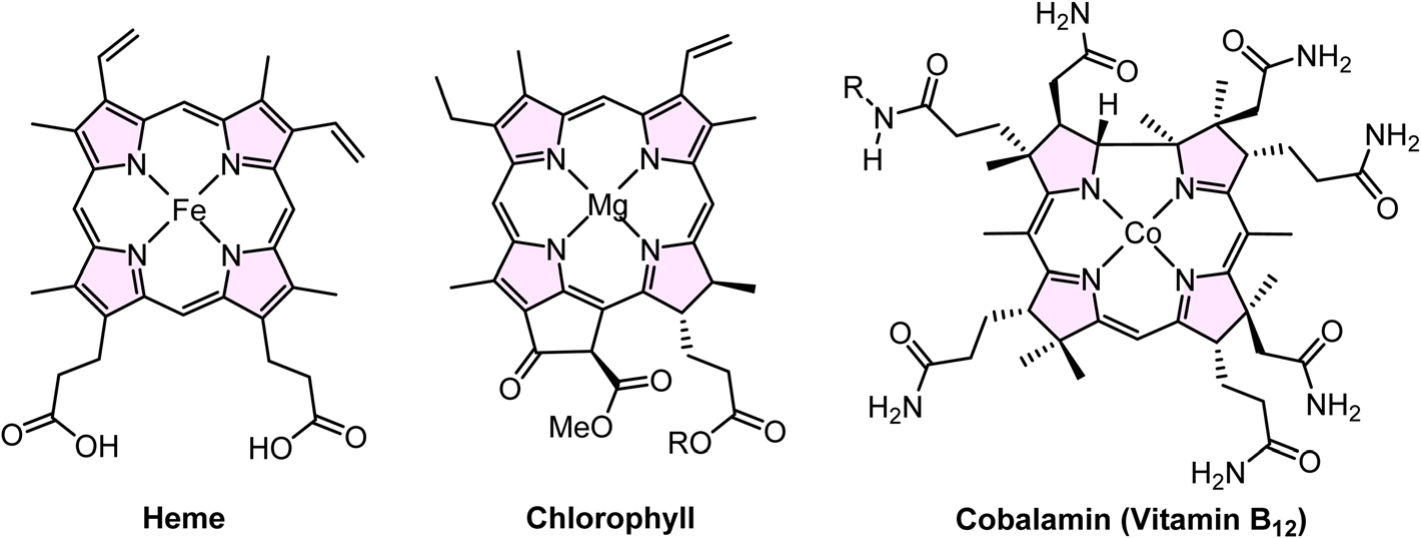
Chemical structures containing the porphyrin ring include heme, chlorophyll and cobalamin (vitamin B12).

Silica's exceptional versatility allows its properties to be tailored for a wide range of industrial applications, including biomedicine, environmental catalysis, agriculture, food processing, chromatography, and pharmaceuticals.^38^ Porphyrins consist of four conjugated pyrrole rings interconnected through methylene bridges at the α-carbon positions, forming a square planar structure.^39^ Substitution of multiple functional groups in the meso position or β-position in the macro porphine cycle produces many porphine derivatives. It is well documented that porphyrins have applications in solar cells,^40–42^ nonlinear optics,^43,44^ molecular electronics,^45^ electrochromism,^46^ imaging,^47^ catalysis,^48^ biomedicine,^49^ chemical sensors,^50^*etc.* Porphyrins' planar and highly symmetrical structure makes them suitable as building blocks in supramolecular assemblies.^51,52^ Moreover, the homogeneously stable configuration of the porphyrin core serves as an ideal site for coordinating various metal ions at the molecular center, resulting in the formation of metal complexes termed metalloporphyrins.^53^ The core structure of porphyrins is also present in the chemical structures of heme, chlorophyll, and cobalamin (Vitamin B_12_) (Fig. 1).^54,55^

The synthesis of artificial porphyrins has garnered substantial attention from researchers, driven by the potential to mimic and manipulate the functionalities observed in these essential biomolecules. Traditional methodologies are amenable for synthesizing porphyrins at a laboratory scale (milligrams).^56,57^ Several established methods have been reported for the synthesis of porphyrins. Rothemund *et al.* demonstrated the synthesis of porphyrin by reacting pyrrole and aldehyde in pyridine at a temperature of 220 °C,^58^ while the one-step Adler–Longo porphyrin synthesis employed acetic or propionic acid as a solvent under aerobic conditions.^59^ Lindsey's two-step method has successfully yielded porphyrin derivatives, ranging from 10% to 60%.^56,57^ These methodologies are associated with several drawbacks, including the requirement for elevated reaction temperatures, the employment of acidic and corrosive conditions, and the resultant low to moderate reaction efficiencies. A significant limitation is the reliance on substantial quantities of chlorinated organic solvents, which poses a substantial barrier to their industrial scalability and application. Also, it is necessary to use DDQ in this method, which ultimately makes the synthetic methodology more expensive.^56,57^

Several syntheses of meso-tetra-aryl porphyrins in the presence of catalysts such as nano-TiCl_4_·SiO_2_,^60^ C_2_H_5_COOH,^61^ BF_3_·OEt_2_,^62^ and HCl^63^ have been reported. These methods have limitations such as synthesis costs, strong acidic conditions, long reaction times, and low yields. Hence, the imperative lies in the development of environmentally benign, cost-effective, and sustainable methodologies for the high-throughput synthesis of porphyrins. To overcome inherent limitations associated with conventional methods, we have developed a novel and straightforward synthetic protocol to produce a series of meso-substituted symmetric A4-porphyrins. In this work, we systematically present the design and development of a silica-modified covalent triazine framework (CTF) as a heterogeneous and effective catalyst for synthesizing A4-porphyrins. Our approach circumvents the reliance on costly oxidants like DDQ and chloranil, opting instead for a mild reaction environment achieved by incorporating TPT-TAT/silica in EtOH. The incorporation of silica onto the CTF substrate plays a crucial role in enhancing the catalytic properties. This modification enhances structural stability and introduces active sites crucial for catalytic activity, with the silica-modified CTF serving as a durable support that optimally immobilizes catalytic species, thereby augmenting the overall catalytic efficiency. The incorporation of an aerobic conditions into the reaction significantly reduces reaction time and enhances efficiency by acting as an oxidizing agent, which accelerates reaction kinetics and improves yields of the desired A4-porphyrins. The synergy between the silica-modified CTF structure and the aerobic conditions creates an optimal environment for the catalytic process, ensuring rapid and efficient synthesis. This approach highlights the potential of silica-modified CTFs in heterogeneous catalysis, offering a promising pathway for the development of efficient and sustainable catalytic systems for various chemical transformations. The findings underscore the importance of structural design and environmental conditions in optimizing catalytic performance.

## Experimental section

### Chemicals and instruments

All chemicals were purchased from Merck and Fluka companies and used without further purifications. ^1^H NMR and ^13^C NMR spectra were run on 500 MHz Bruker spectrometer and have been reported in DMSO-*d*_6_ with ppm chemical displacement. Shimadzu 435-U-04 FT spectrophotometer was used to record FT-IR spectra. The HR-Mass spectrometer was recorded in a spectrometer Bruker Microflex LT MALDI-TOF MS. Field Emission Scanning Electron Microscopy (FE-SEM) was recorded using the MIRA III device model. High-Resolution Transmission Electron Microscopy (HR-TEM) was performed by FEI TECNAI F20. Brunauer–Emmett–Teller (BET) analysis was recorded by BELSORP Mini II. Energy dispersive X-ray (EDX) was fulfilled on a MIRA II (France) instrument. X-ray diffraction (XRD) was recorded by Philips PW1730. Thermogravimetric analysis (TGA) was accomplished on a Q600, (America) Instruments.

### Preparation of *N*^2^,*N*^4^,*N*^6^-tris(4-(aminomethyl) benzyl)-1,3,5-triazine-2,4,6-triamine

Cyanuric chloride (1 mmol, 0.184 g) was introduced into a flask, which was then filled with 10 mL of acetone and placed in an ice bath. Following this, a solution composed of 10 mL of water and ice was added, producing a white suspension. 1,4-phenylenedimethanamine (3.2 mmol, 0.43 g) and sodium hydroxide (3.2 mmol, 0.128 g) were dissolved in an acetone/water solution (1 : 1) and alternately added to the suspension. The mixture was refluxed for 2 hours at 0 °C and stirred 2 hours at room temperature, and then at 60 °C for 18 hours. The reaction progress was monitored using thin layer chromatography (TLC). Upon completion of the reaction, the formed precipitates were filtered and dried in oven at 100 °C. The structure of the synthesized product was determined by FT-IR, NMR and HR-Mass techniques.

### Preparation of 2,4,6-tris(4-formylphenoxy)-1,3,5-triazine

This compound was prepared according to the previously reported method.^64^ Cyanuric chloride (1 mmol, 0.184 g) was poured into a 50 mL flask and 10 mL of acetone was added. After that, the flask was placed in an ice bath. A white suspension was obtained by adding 10 mL of water and ice mixture to the solution. Then *p*-hydroxybenzaldehyde (3.2 mmol, 0.39 g) and sodium hydroxide (3.2 mmol, 0.128 g) were dissolved in a solution containing acetone/water (1 : 1) and added drop by drop to the resulting suspension. The resulting mixture was stirred at 0 °C for 2 hours, then at room temperature for 2 hours, and finally refluxed at 60 °C for 18 hours. The progress of the reaction was followed by thin layer chromatography (TLC). After the completion of the reaction, the precipitates formed were filtered. The sediments were washed with cold water and dried at 100 °C. The structure of the final product was confirmed by FT-IR, NMR and HR-Mass techniques.

### Synthesis of covalent triazine framework (TPT-TAT)

2,4,6-Tris(4-formylphenoxy)-1,3,5-triazine (1 mmol, 0.441 g), *N*^2^,*N*^4^,*N*^6^-tris(4-(aminomethyl)benzyl)-1,3,5-triazine-2,4,6-triamine (1 mmol, 0.483 g), 1,4-dioxane (30 mL) and acetic acid (3 M, 0.6 mL) were combined in a pyrex tube. The tube was placed in an ultrasonic bath for 1 h, then placed in a liquid N_2_ bath at 77 K and degassed three times through a freeze–pump–thaw cycle. The reaction contents were transferred in a Teflon-lined autoclave stainless steel reactor and placed in an oven at 120 °C for 8 days. The yellow precipitates obtained were collected by filtration and then washed with *N*,*N*-dimethylformamide (3 × 10 mL) and tetrahydrofuran (3 × 10 mL). The resulting powder was dried at 80 °C for 24 hours in a vacuum oven.

### Anchoring of silica on covalent triazine framework (TPT-TAT/silica)

**Scheme 1:**
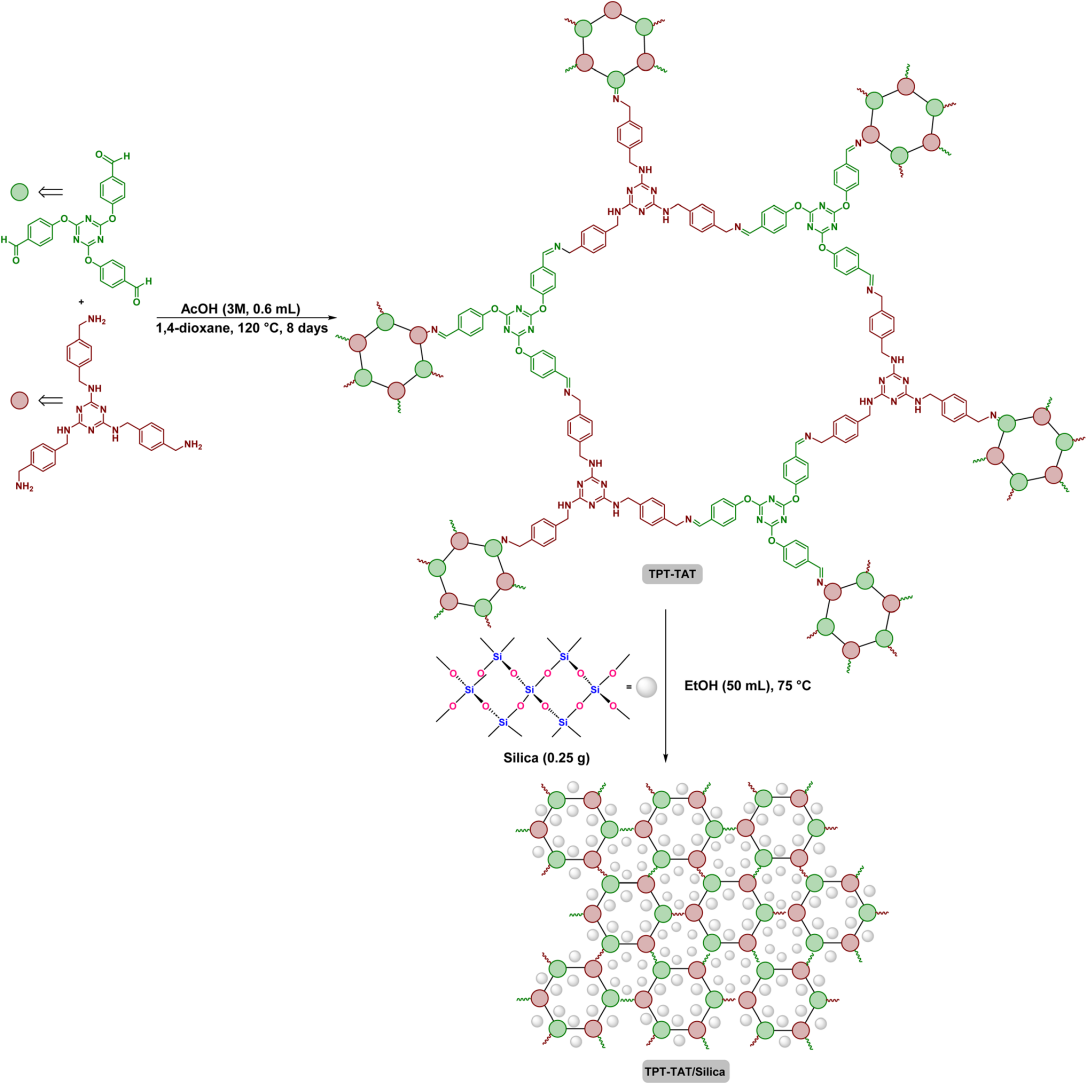
Structural representation of the TPT-TAT/silica, illustrating key interactions and features.

First, silica (0.25 g) was dissolved in EtOH (50 mL) and added to TPT-TAT (0.5 g). The mixture was incubated at 75 °C for 2 h, then centrifuged and placed in a vacuum oven to dry (Scheme 1).

### General synthesis of *meso*-substituted symmetric A4-porphyrins

Pyrrole (4 mmol, 0.28 mL), benzaldehyde (4 mmol, 0.4 mL), TPT-TAT/silica (0.045 g), and EtOH (40 mL) were added to a round-bottom flask. The reaction mixture was stirred in the dark at 80 °C for 4 h under aerobic conditions. The color of the mixture changed to dark purple. The catalyst was separated from the reaction mixture by centrifugation. Column chromatography using a mixture of *n*-hexane and dichloromethane (40 : 10) was employed to purify the product. This solvent mixture was selected for its ability to effectively separate the desired porphyrin product from unreacted starting materials and by-products based on their differing polarities. The comprehensive characterization of the obtained product was performed using FT-IR, HR-Mass, and NMR spectroscopy.

## Results and discussion

**Fig. 2:**
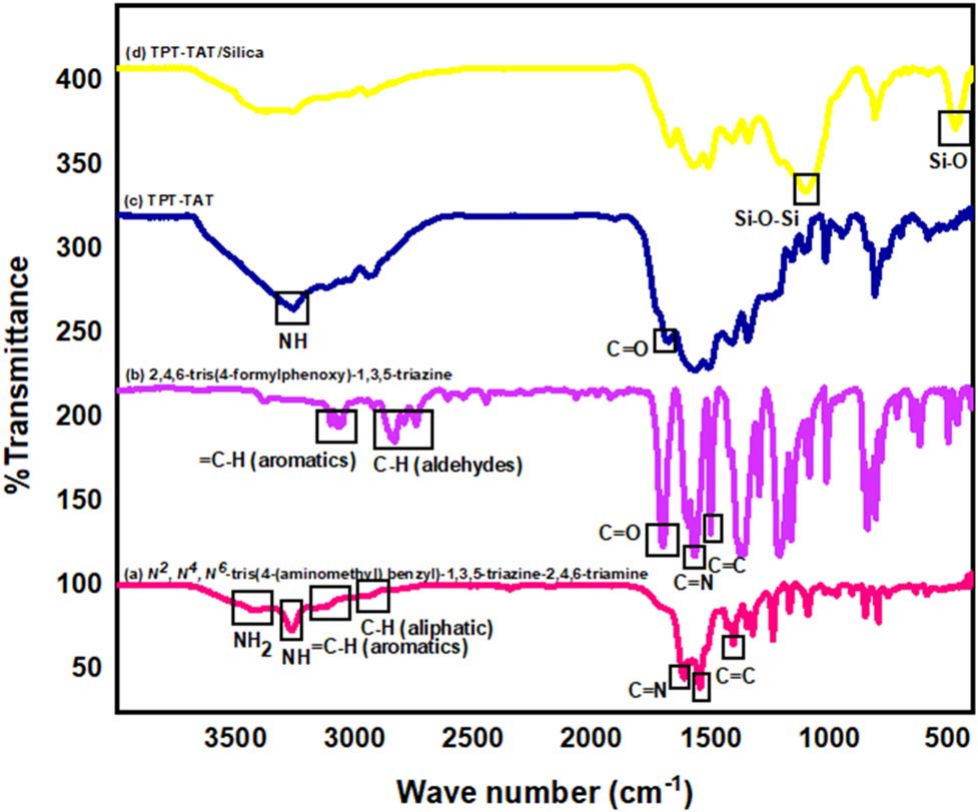
FT-IR spectrum of raw and intermediate materials and the final structure of the catalyst: (a) *N*^2^,*N*^4^,*N*^6^-tris(4-(aminomethyl) benzyl)-1,3,5-triazine-2,4,6-triamine, (b) 2,4,6-tris(4-formylphenoxy)-1,3,5-triazine, (c) TPT-TAT, (d) TPT-TAT/silica.

The polymerization process of the covalent triazine framework (CTF) involves the condensation of two key precursors include, *N*^2^,*N*^4^,*N*^6^-tris(4-(aminomethyl) benzyl)-1,3,5-triazine-2,4,6-triamine and 2,4,6-tris(4-formylphenoxy)-1,3,5-triazine. This reaction is conducted under solvothermal conditions at 120 °C for 8 days in a sealed environment, which facilitates the formation of the CTF through the creation of covalent bonds between the precursors. Fourier transform infrared spectroscopy (FT-IR) was used to characterize the starting materials, CTF and CTF/silica (Fig. 2). The following key peaks observed in the FT-IR spectra are crucial for understanding the polymerization mechanism.

In the FT-IR spectrum of *N*^2^,*N*^4^,*N*^6^-tris(4-(aminomethyl) benzyl)-1,3,5-triazine-2,4,6-triamine (Fig. 2a), the peaks in the region of 3422 and 3400 cm^−1^ are related to NH_2_ group. The peak index at 3265 cm^−1^ is specific to the NH group. The C–H stretching vibration of aromatics has appeared in 3052 and 3011 cm^−1^. The stretching vibration in the 2923 and 2918 cm^−1^ region is associated with aliphatic moieties. The stretching vibration of the C=N group appeared at 1615 cm^−1^ and the peaks at 1546, and 1405 cm^−1^ are attributed to the aromatic C=C stretching vibration. In the FT-IR spectrum of 2,4,6-tris(4-formylphenoxy)-1,3,5-triazine (Fig. 2b), the absorption bands at 3102 and 3170 cm^−1^ are related to C–H aromatic stretching vibration. The characteristic peaks at 2739 and 2834 cm^−1^ are specific to C–H stretching vibration of aldehyde. The sharp peak in the 1703 cm^−1^ is attributed to the C=O stretching vibration. The C=N stretching vibration appeared in 1567 cm^−1^ and the C=C aromatic stretching vibration was identified in 1502 cm^−1^. In the FT-IR spectrum of CTF (Fig. 2c), although the absorption band of the new C=N bond resulting from the condensation of aldehyde and amine cannot be identified, the reduction of the NH_2_ peak indicates a significant level of polymerization. The stretching vibration of 1679 cm^−1^ can be attributed to the terminal aldehyde at the edges of CTF. This result shows the formation of an extensive framework of density. In the FT-IR spectrum of CTF/silica (Fig. 2d), the stretching vibration appearing at 1099 cm^−1^ and 471 cm^−1^ is related to Si–O–Si and Si–O, respectively. These results show that silica is successfully immobilized on CTF. This polymerization process results in a highly porous framework with elevated nitrogen content, which is essential for creating catalytic active sites. The resulting CTF demonstrates exceptional catalytic performance in subsequent applications, such as porphyrin synthesis.

**Fig. 3:**
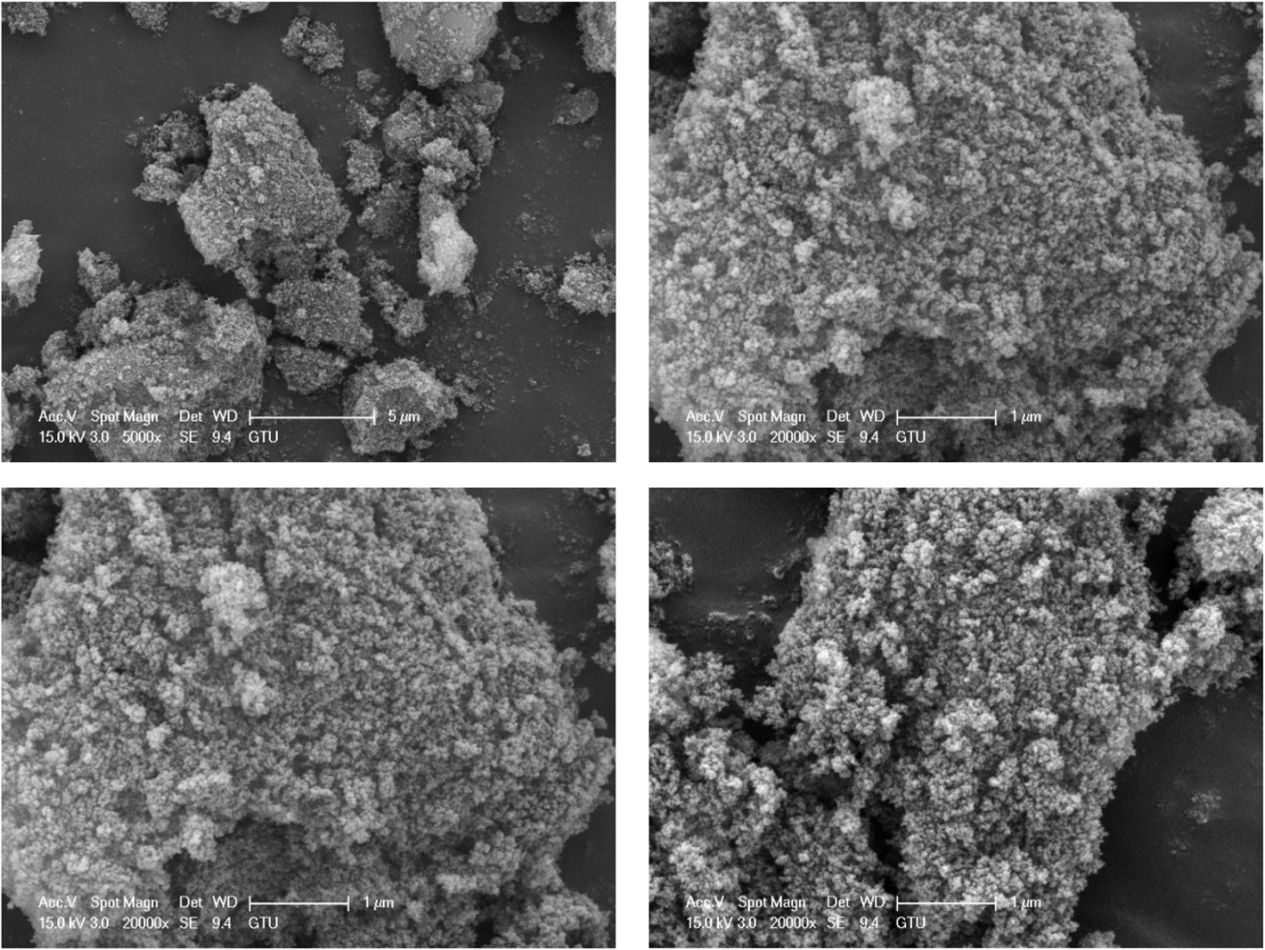
Scanning electron microscopy (SEM) images of the TPT-TAT/silica.

Surface morphology analysis using field emission scanning electron microscopy (FE-SEM) provided comprehensive insights into the structural characteristics of TPT-TAT/silica particles, as depicted in Fig. 3. This advanced imaging technique allowed for high-resolution visualization of the surface features, enabling a detailed examination of the particle morphology. The FE-SEM results revealed that TPT-TAT exhibits a predominantly spherical structure. This spherical morphology is indicative of uniform particle formation. Moreover, the analysis showed that silica is effectively stabilized on the surface of these spherical TPT-TAT particles. The presence of silica on the surface suggests successful modification and integration, which can enhance the material's stability and functionality. Silica stabilization may also provide additional active sites and improve the material's resistance to degradation under different environmental conditions. Overall, the FE-SEM analysis not only confirmed the spherical structure of TPT-TAT but also demonstrated the successful stabilization of silica on its surface.

**Fig. 4:**
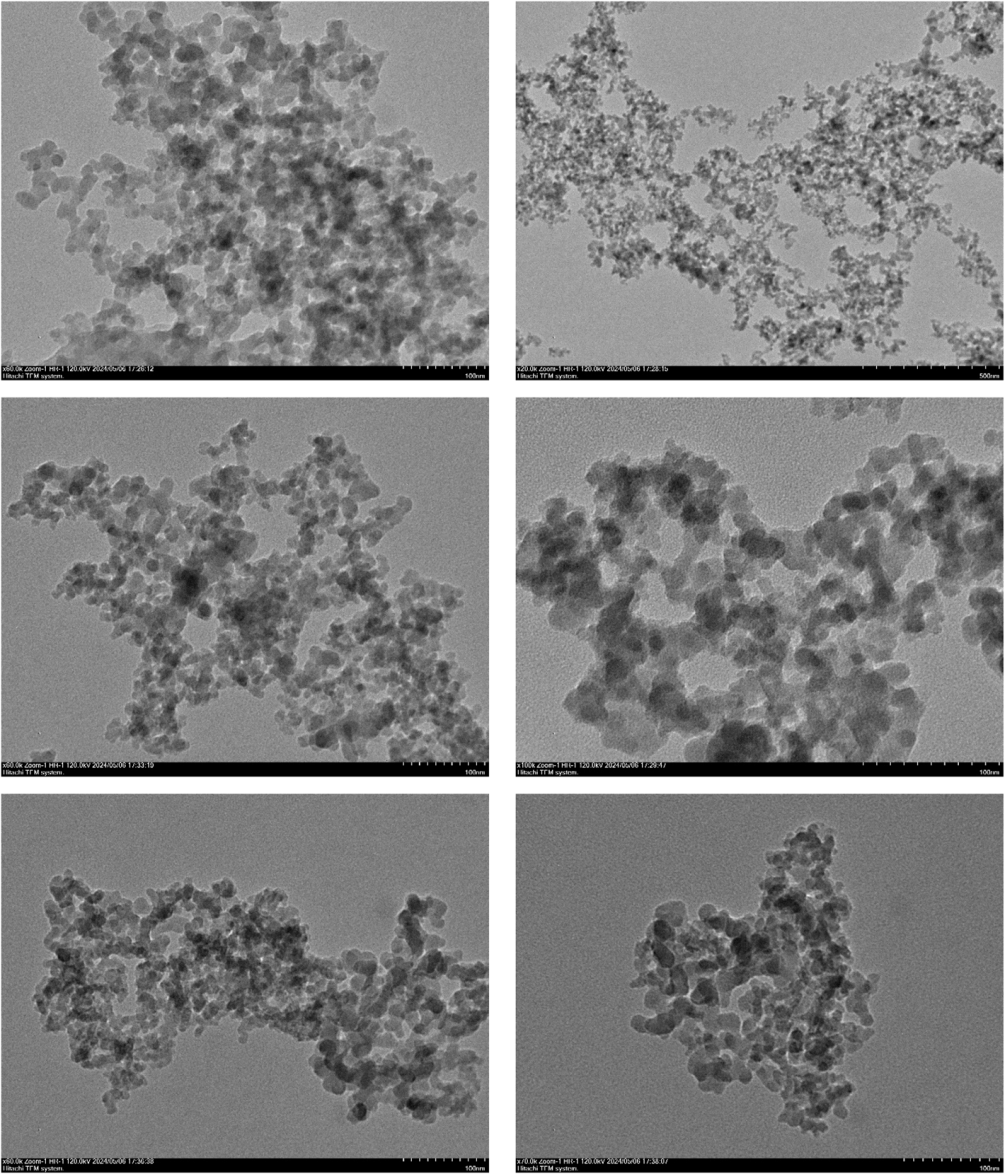
Transmission electron microscopy (TEM) images of the TPT-TAT/silica.

Transmission electron microscopy (TEM) analysis was performed to investigate the microstructure of the covalent triazine framework. The high-resolution TEM images showed the ordered arrangement of the triazine units, confirming the formation of a well-defined and interconnected network within the framework. Additionally, TEM analysis provided valuable information about the particle size distribution, surface characteristics, and overall structural integrity of the covalent triazine framework (Fig. 4).

**Fig. 5:**
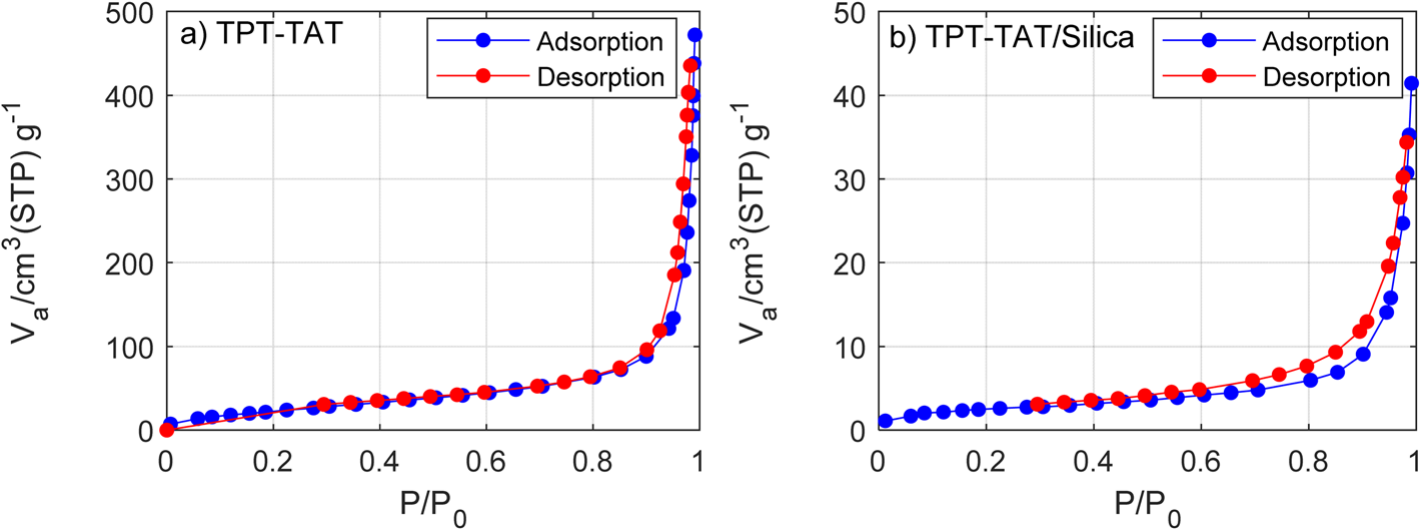
Adsorption and desorption isotherms of (a) TPT-ATA and (b) TPT-TAT/silica. Adsorptive N_2_ and adsorption temperature at 77 K.

The porosity and surface areas TPT-TAT and TPT-TAT/silica were measured by nitrogen adsorption–desorption analysis at 77 K. Fig. 5 displays the adsorption and desorption isotherms of TPT-ATA (a) and TPT-TAT/silica (b). Adsorptive N_2_ and adsorption temperature at 77 K. The Brunauer–Emmett–Teller (BET) indicates surface areas (*S*) of 93.739 and 9.2927 m^2^ g^−1^ for TPT-TAT and TPT-TAT/silica, respectively. The reduced surface area in TPT-TAT/silica compared to TPT-TAT is attributed to the binding of silica particles to the TPT-TAT surface. The calculated total pore volume (*V*_P_) in *P*/*P*_0_ = 0.99 is 0.00246 cm^3^ g^−1^ for TPT-TAT. Also, based on the BET analysis the mean pore diameter (*r* = 2*V*_P_/*S*) for TPT-TAT and TPT-TAT/silica, respectively, are 29.801 and 26.474 nm. The reduction in N_2_ uptake and surface area in TPT-TAT/silica is attributed to the stabilization of silica. Barrett–Joyner–Halenda model (BJH) analysis revealed 1.21 and 2.1 nm pore widths for TPT-TAT and TPT-TAT/silica, respectively.

The incorporation of silica into the covalent triazine framework (CTF) plays a significant role in enhancing the material's properties, despite the observed decrease in overall surface area. The modification with silica can create new active sites on the CTF surface that are not solely dependent on surface area metrics. These active sites may arise from the interaction between silica and CTF can lead to unique catalytic sites that facilitate specific reactions. Silica can improve the accessibility of active sites within the CTF by altering pore sizes or creating microenvironments conducive to catalysis. Silica can improve the accessibility of active sites within the CTF by altering pore sizes or creating microenvironments conducive to catalysis. The silica is strategically placed within the cavities of the CTF, which contributes to several beneficial effects. The presence of silica reinforces the structural integrity of the CTF, preventing collapse or degradation during catalytic processes. This stabilization is crucial for maintaining the framework's porosity and functionality over time. While the total surface area may decrease, silica can create localized active sites at the interface between the silica and CTF. These sites can enhance catalytic activity by providing additional points for reactant interaction, even if they are not fully reflected in surface area measurements. Also, the silica support improves the material's resistance to leaching and physical degradation during reaction cycles. This characteristic is particularly important for maintaining catalytic efficiency and facilitating easy recovery of the catalyst after reactions. In addition, the combination of CTF and silica creates an optimal environment for catalysis. The unique properties of both materials can synergistically enhance reaction kinetics and improve yields in processes such as porphyrin synthesis.

**Fig. 6:**
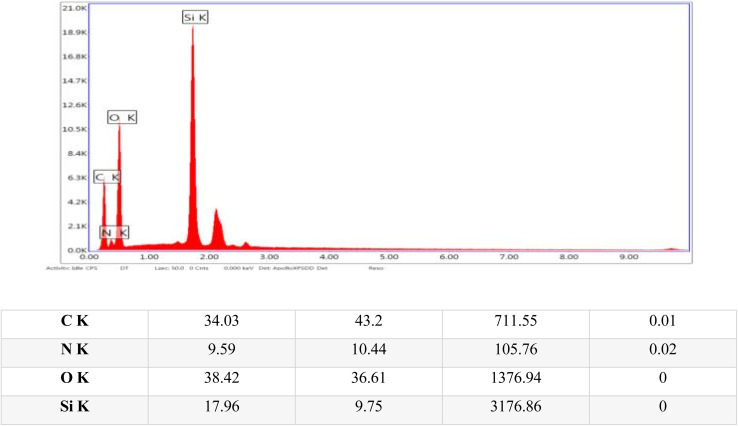
Energy dispersive X-ray (EDX) images of the TPT-TAT/silica, illustrating elemental distribution and composition.

Energy-dispersive X-ray (EDX) analysis was conducted to identify and quantify the elements in the sample, thereby providing detailed insights into its elemental composition and chemical makeup. The EDX analysis confirmed the presence of several key structural elements within the sample, including carbon, oxygen, nitrogen, and silicon, as illustrated in Fig. 6.

**Fig. 7:**
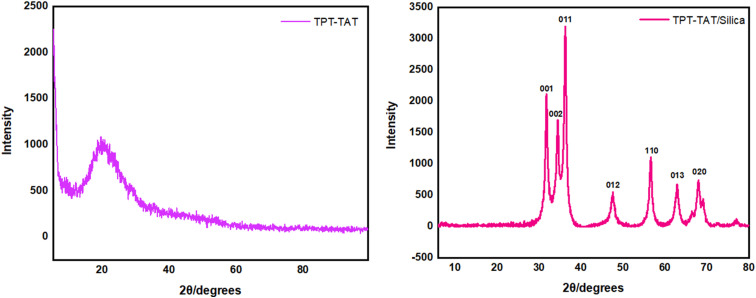
X-ray diffraction (XRD) pattern of the TPT-TAT/silica.

Analysis of the X-ray powder diffraction (XRD) patterns for TPT-TAT, as shown in Fig. 7, revealed a prominent diffraction peak at 2*θ* = 20.29, indicating an amorphous morphology of the material.^65^ This characteristic peak suggested the absence of long-range order or crystalline phases within the material. XRD analysis of TPT-TAT/silica at 2*θ* = 31.7, 34.4, 36.2, 47.5, 56.6, 62.8, and 68.0 refer to the (001), (002), (011), (012), (110), (013), and (020) reveals characteristic peaks, indicative of crystalline silica nanoparticles.^66^

**Fig. 8:**
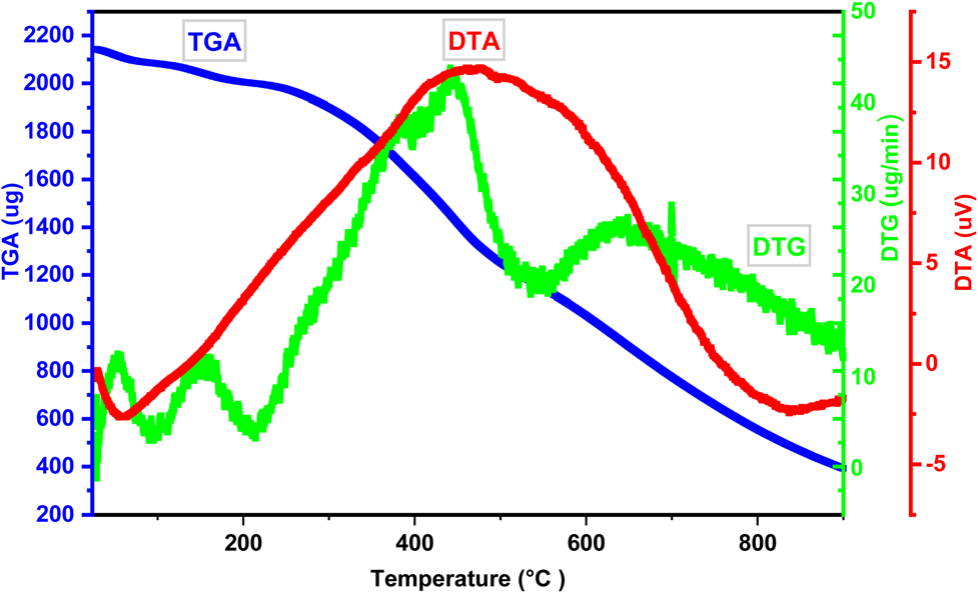
Thermogravimetric (TG), differential thermal analysis (DTA), and derivative thermogravimetric (DTG) graph of the TPT-TAT/silica, illustrating thermal stability and decomposition behavior.

Thermal stability was assessed using thermogravimetric analysis (TGA), conducted at a heating rate of 10 °C min^−1^ under a nitrogen atmosphere, over a temperature range of 25 to 900 °C. DTG analysis of the TGA data reveals four distinct weight loss steps, with the initial weight loss observed between 25 and 152 °C attributed to the removal of water and organic solvents. The DTA and DTG diagrams show the major destruction of the catalyst structure at 480 °C, which indicates the destruction of the bonds in the sample. The final decomposition of the catalyst structure occurred at 650 °C. The results show the high thermal stability of the catalyst (Fig. 8).

### TPT-TAT/silica catalytic activity

This section investigates the catalytic efficacy of TPT-TAT/silica in porphyrin synthesis, focusing on the condensation reaction between pyrrole and benzaldehyde under a range of conditions, including variations in solvent types, catalyst loadings, and reaction temperatures (Table 1, entries 1–15). To evaluate the catalytic performance and determine the optimal conditions, the model reaction was conducted under various scenarios including different solvents, temperatures, and catalyst loadings and the optimal yield was achieved when employing ethanol as the solvent with 0.045 g of catalyst at a reaction temperature of 80 °C (Table 1, entry 10).

**Table 1:** Optimization of the reaction conditions for the synthesis of symmetric A4-porphyrins^a^

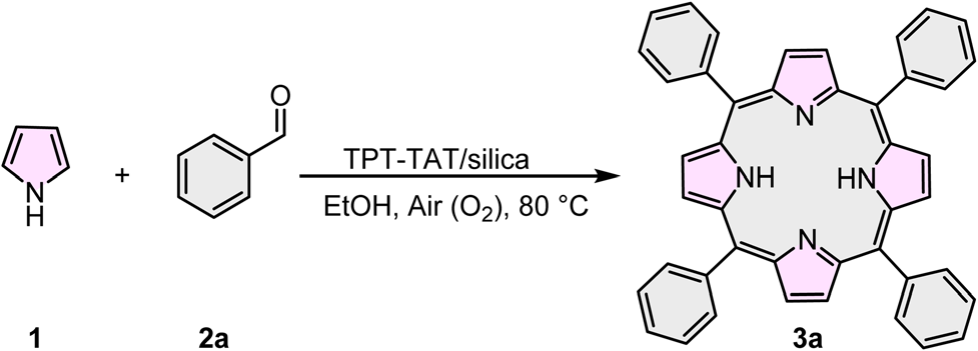					
Entry	Catalyst (g)	Solvent	Time (h)	*T* (°C)	Yield^b^ (%)
1	—	—	4	75	N.R.
2	—	EtOH	4	75	N.R.
3	0.01	EtOH	6	30	N.R.
4	0.045	EtOH	6	30	16
5	0.1	EtOH	6	30	21
6	0.01	EtOH	5	55	13
7	0.045	EtOH	4	55	20
8	0.1	EtOH	4	55	32
9	0.01	EtOH	4	80	41
10	0.045	EtOH	4	80	81
11	0.1	EtOH	4	80	81
12	0.045	H_2_O	4	90	N.R.
13	0.045	DMF	4	90	N.R.
14	0.045	CH_3_CN	4	80	16
15	0.045	—	4	80	10

a Reaction conditions: pyrrole (4 mmol), benzaldehyde (4 mmol), catalyst (0.045 g), EtOH (40 mL).

b Isolated yields.

Subsequent to optimizing the model reaction, we examined the effect of TPT-TAT/silica in combination with pyrrole 1 and a series of aldehydes 2(a)–(h). The results demonstrated that both electron-withdrawing and electron-donating aldehydes significantly enhanced the efficiency of the desired product's formation (Table 2).

**Table 2:** Synthesis of various symmetric A4-porphyrins^a^

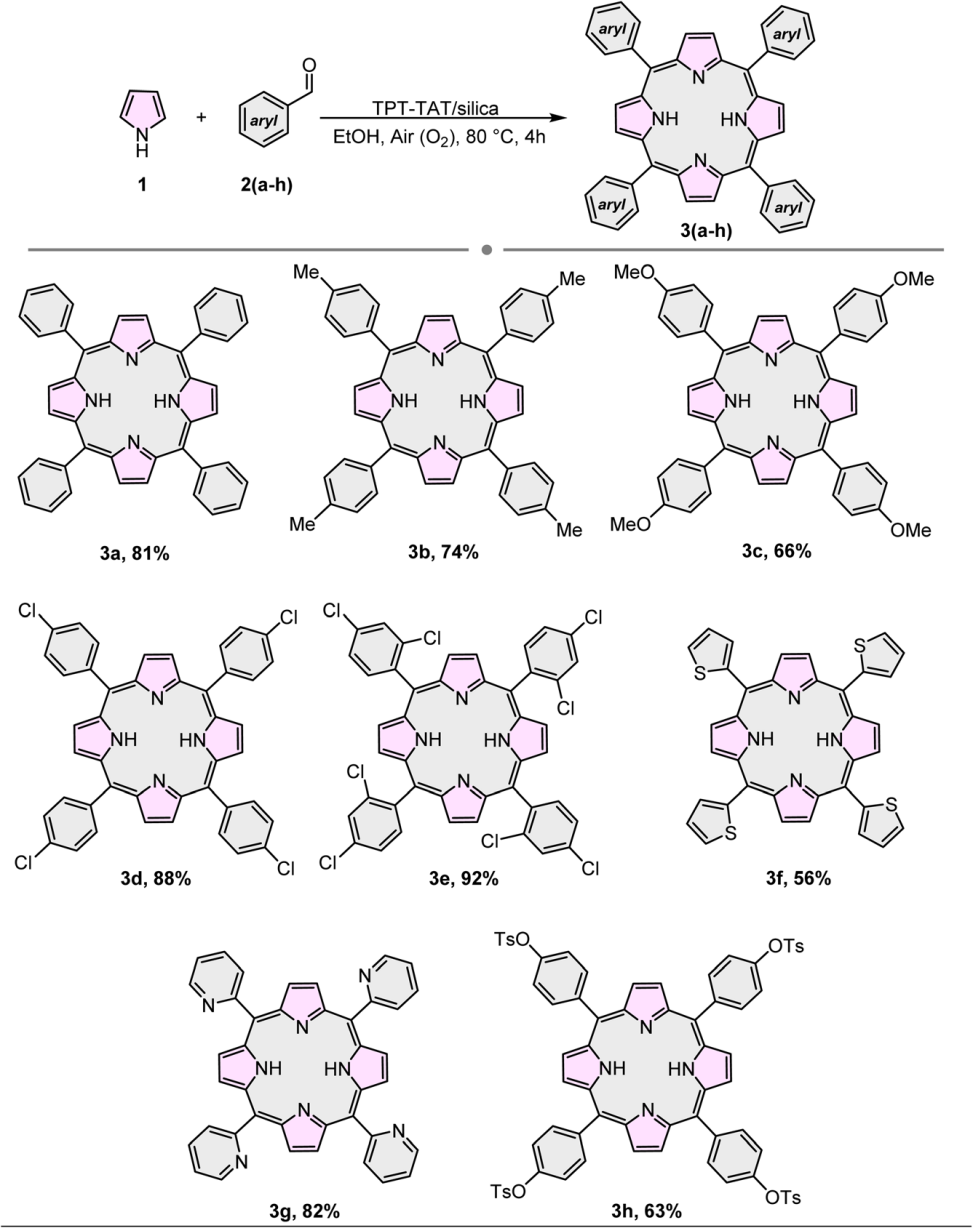

a Reaction conditions: pyrrole (4 mmol), benzaldehydes (4 mmol), catalyst (0.045 g), EtOH (40 mL).

In this study, TPT-TAT/silica was employed for the synthesis of porphyrin derivatives, offering a notable advantage by avoiding harsh acidic conditions, which distinguishes it from prior methods. Furthermore, the exclusion of costly oxidants and the use of ethanol as a solvent underscore the method's alignment with green chemistry principles, highlighting its environmental and economic benefits. To evaluate the efficiency of the reaction process, a complementary experiment was conducted using a model reaction with TPT-TAT and silica, as detailed in Table 3. This experiment was designed to compare the catalytic performance of the TPT-TAT/silica system with that of TPT-TAT and silica used individually. The results obtained from this study clearly demonstrated the superior performance of the TPT-TAT/silica combination. Specifically, this system exhibited a significantly reduced reaction time, indicating a faster reaction rate compared to alternatives. Moreover, the efficiency of the reaction, as measured by yield or conversion rate, was notably higher when TPT-TAT/silica was used. These findings underscore the potential of TPT-TAT/silica as an effective catalyst system, offering both time-saving and efficiency advantages in the model reaction.

**Table 3:** Comparison of catalyst completion steps for the synthesis of A4-porphyrins

Entry	Catalyst	Time (h)	Yield (%)
1	TPT-TAT	4	45
2	Silica	4	Trace
3	TPT-TAT/silica	4	81

The proposed method was benchmarked against established procedures detailed in the literature and the results demonstrate that it achieves significantly higher efficiency in porphyrin synthesis compared to the reported methods (Table 4). The catalyst demonstrated effective utilization of its active sites, significantly enhancing the reaction rate and overall efficiency of the porphyrin synthesis process. Moreover, the substitution of costly oxidants with an aerobic condition as the oxidizing agent and the use of ethanol as a green solvent enhance the reaction's environmental sustainability. The results indicate that TPT-TAT/silica exhibits a substantial improvement in efficiency and sustainability compared to other methods in porphyrin synthesis; furthermore, this innovative catalyst's insoluble nature bestows upon it a remarkable advantage, allowing for facile separation from the reaction mixture and subsequent reuse.

**Table 4:** Comparison of various catalyst in synthesizing porphyrin derivatives

Entry	Catalyst (g)	Oxidant	Solvent	Temp. (°C)	Time (h)	Yields (%)	Ref.
1	PCl_5_ (0.04)	Air	CH_2_Cl_2_	Reflux	4	62	67
2	CF_3_SO_2_Cl (0.1 mL)	Air	CH_2_Cl_2_	Reflux	4	65	68
3	SSA (0.05)	Air	CH_2_Cl_2_	rt	4	60	69
4	Nano-TiCl_4_·SiO_2_ (0.1)	CAN/Air	CH_2_Cl_2_	rt	1	80	60
5	TPT-TAT/silica (0.045)	Air	EtOH	80	4	81	Our work

### Recycling and reusing the catalyst

**Fig. 9:**
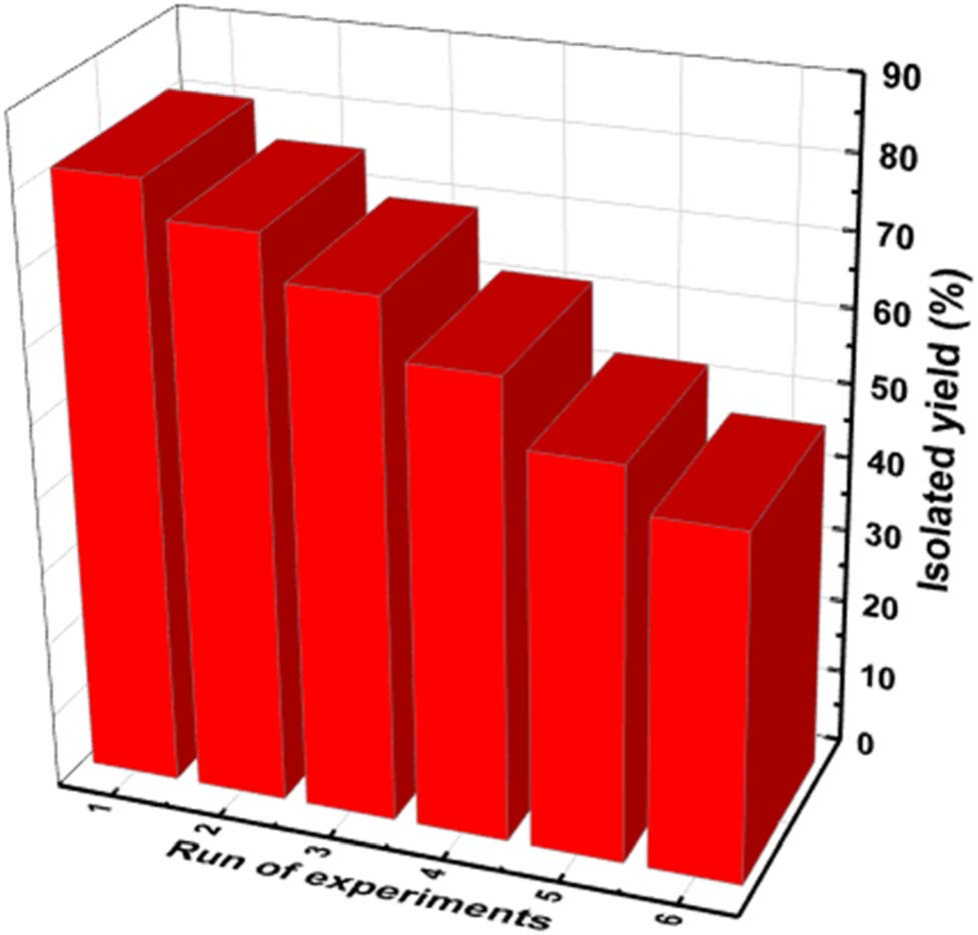
Assessment of catalyst reusability for the TPT-TAT/silica composite, demonstrating performance across multiple reaction cycles.

To assess the recyclability of the catalyst, a model reaction involving pyrrole and benzaldehyde was conducted using 0.045 g of the catalyst. After each reaction cycle, the catalyst was recovered through centrifugation, followed by washing with hot ethanol. Once dried, the catalyst was reused in subsequent reactions. The findings demonstrated that the catalyst retained its activity over six cycles with minimal degradation in performance (Fig. 9).

**Fig. 10:**
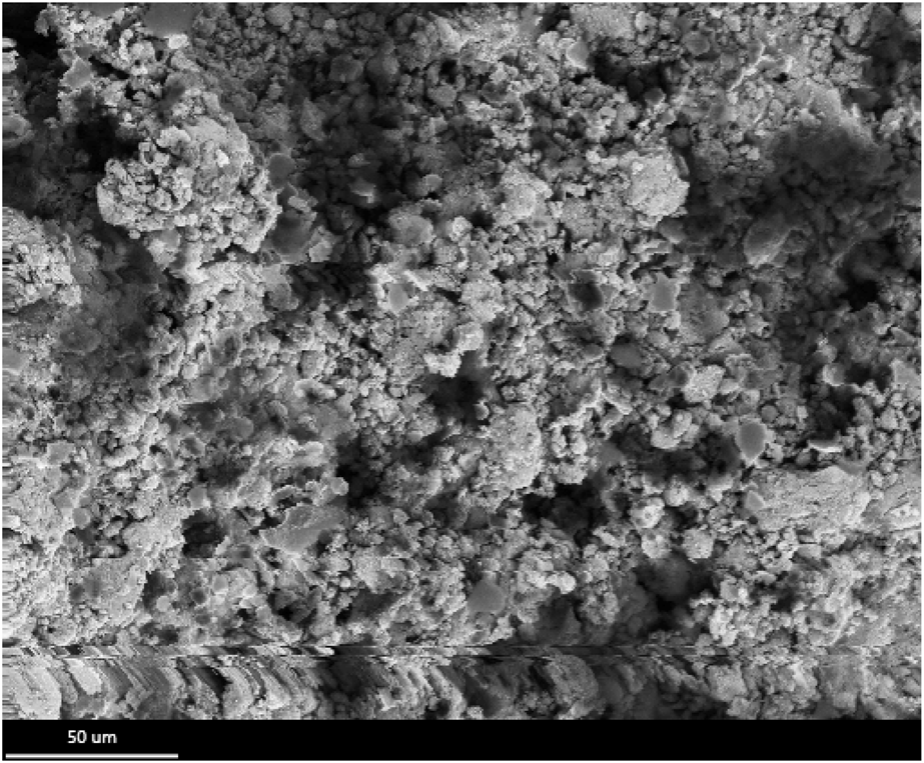
SEM image of TPT-TAT/silica after the recycling.

The field emission scanning electron microscopy (FE-SEM) analysis was conducted on the novel silica stabilized on covalent triazine framework frame after multiple recycling cycles. The results revealed that the catalyst's structure remained intact and unchanged, even after several uses. This observation indicates the remarkable stability of the catalyst, demonstrating its potential for repeated applications without significant structural degradation (Fig. 10).

**Fig. 11:**
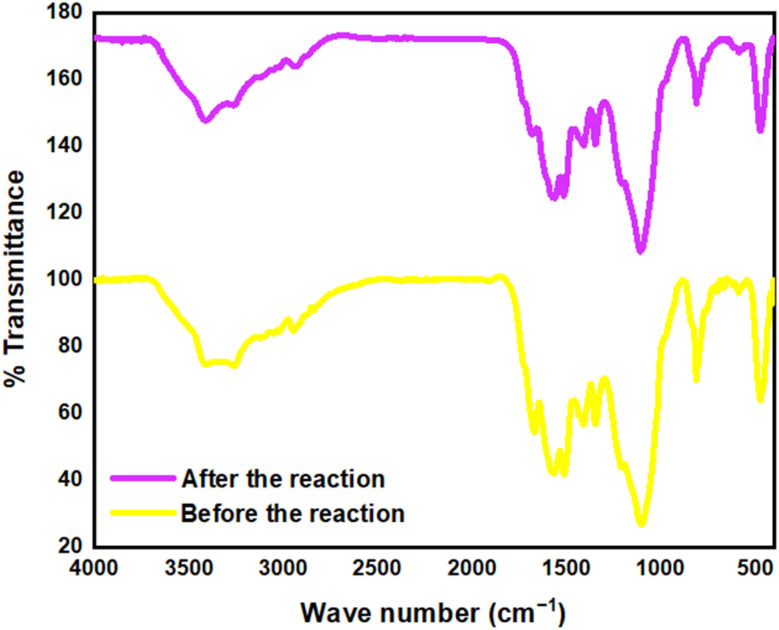
FT-IR spectra of TPT-TAT/silica before and after the reaction.

The Fourier-Transform Infrared (FT-IR) spectroscopy analysis was conducted on the silica stabilized on covalent triazine framework after several cycles of use. The results revealed that the catalyst's structure remained stable, with no significant changes observed in the spectral patterns. This finding indicates the remarkable structural integrity of the catalyst, even after multiple reaction cycles (Fig. 11).

### The proposed reaction mechanism

**Scheme 2:**
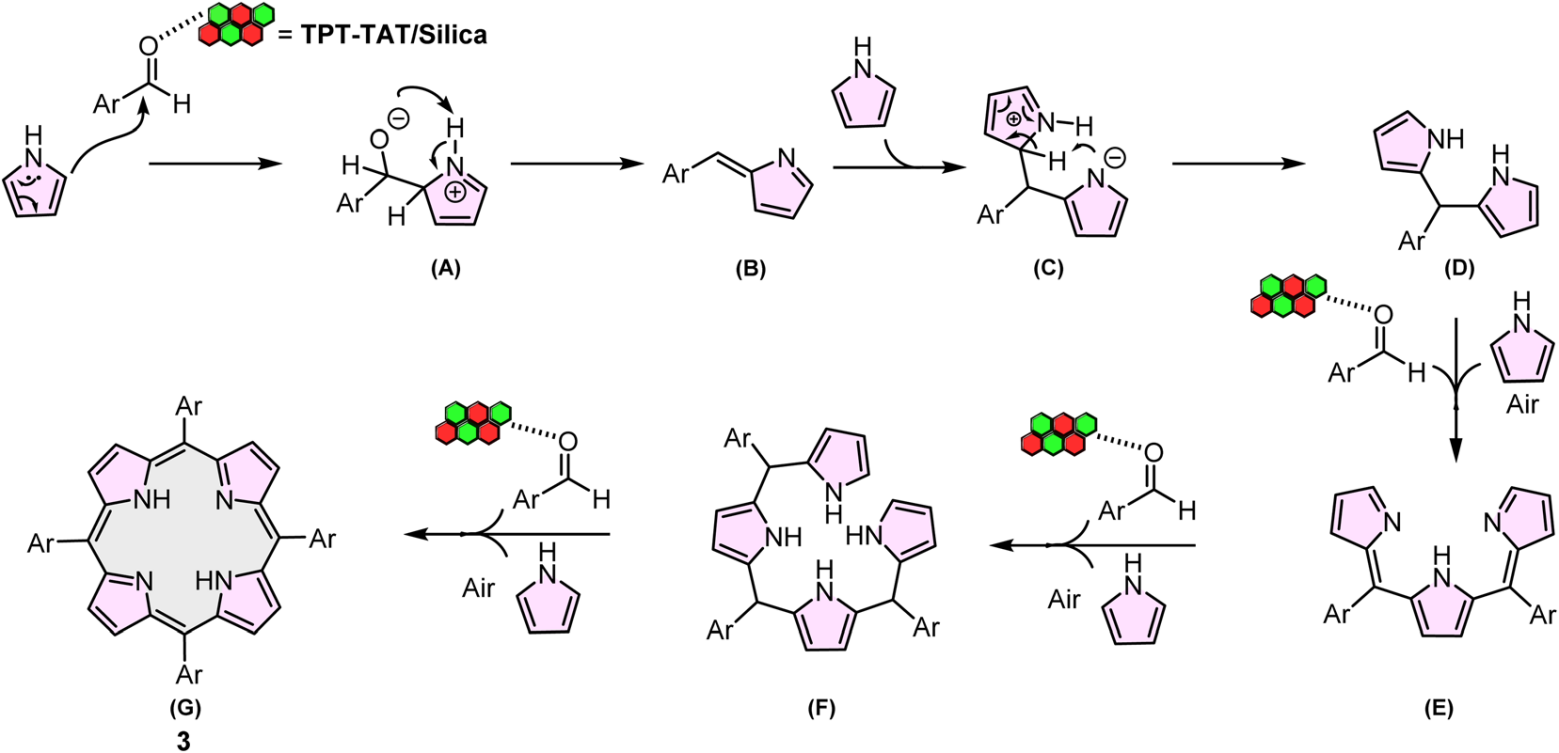
Proposed mechanism for the synthesis of A4-porphyrin.

A proposed mechanistic pathway for the synthesis of A4-porphyrin derivatives 3 is outlined in Scheme 2. The mechanism commences with the electrophilic substitution of the activated aldehyde by TPT-TAT/silica at the α-pyrrole position, resulting in the formation of intermediate A. Subsequently, the nitrogen in the TPT-TAT/silica structure abstracts a hydrogen atom from the pyrrole, yielding intermediate B. The reaction of intermediate B with pyrrole produces intermediate C. The negatively charged nitrogen in intermediate C then abstracts an α-hydrogen from an adjacent pyrrole ring, forming intermediate D. This sequence of steps continues, leading to the formation of intermediates E and F. Finally, intermediate F reacts with an aldehyde to produce the porphyrin product (G).^70^

## Conclusions

In summary, we synthesized novel covalent triazine frameworks (CTFs) by condensing 2,4,6-tris(4-formylphenoxy)-1,3,5-triazine with *N*^2^,*N*^4^,*N*^6^-tris(4-(aminomethyl) benzyl)-1,3,5-triazine-2,4,6-triamine. The resulting CTF exhibited a high specific surface area, exceptional thermal stability, a well-defined porous structure, and substantial nitrogen content, rendering it highly suitable for the immobilization of silica. The CTF/silica composite demonstrated superior catalytic activity, facilitating the efficient synthesis of various porphyrin derivatives in a shortened reaction time. Moreover, the catalyst showed excellent reusability, maintaining its performance across multiple cycles and providing a practical and sustainable option for catalytic applications.

## Spectral data of compounds

### Compound 1a

^1^H NMR (250 MHz, DMSO-*d*_6_) *δ* 9.96 (s, 3H), 7.95 (d, *J* = 8.4 Hz, 6H), 7.47 (d, *J* = 8.4 Hz, 6H). ^13^C NMR (63 MHz, DMSO) *δ* 192.34, 173.14, 156.05, 134.51, 131.59, 122.78, 40.93. HR-Mass (*m*/*z*): 441.29.

### Compound 2a

^1^H NMR (250 MHz, DMSO-*d*_6_) *δ* 10.35 (s, 6H), 9.62 (s, 3H), 7.27 (d, *J* = 11.9 Hz, 12H), 4.61 (s, 6H), 4.47 (s, 7H). ^13^C NMR (63 MHz, DMSO) *δ* 165.84, 154.19, 136.95, 127.89, 44.13, 43.81. HR-Mass (*m*/*z*): 483.62.

### Compound 3a

^1^H NMR (500 MHz, NONE) *δ* 10.39, 7.19, 6.08, 5.00, 4.06.

### 5,10,15,20-Tetraphenylporphyrin (4a)

81% yield; FT-IR (KBr, *ν*, cm^−1^): 3425, 3023, 2925, 2852, 1675, 1570, 1451. ^1^H NMR (400 MHz, DMSO-*d*_6_) *δ* 7.94 (s, 8H), 7.53 (t, *J* = 8.0, Hz, 8H), 7.23 (d, *J* = 4.3 Hz, 8H), 7.12 (t, *J* = 4 Hz, 4H), −2.76 (s, 2H). HR-Mass (*m*/*z*): 614.75 [M + H]^+^, found: 614.723 [M + H]^+^.^71^

### 5,10,15,20-Tetrakis(4-methylphenyl)porphyrin (4b)

74% yield; FT-IR (KBr, *ν*, cm^−1^): 3434, 3096, 3017, 2919, 2859, 1689, 1577, 1510. ^1^H NMR (400 MHz, DMSO-*d*_6_) *δ* 8.09 (s, 8H), 7.02 (d, *J* = 8.0 Hz, 8H), 6.93 (d, *J* = 7.3 Hz, 8H), 2.20 (s, 12H), −2.76 (s, 2H). ^13^C NMR (126 MHz, DMSO-*d*_6_) *δ*^13^C NMR (126 MHz, DMSO) *δ* 141.57, 135.21, 133.19, 128.79, 128.48, 106.05, 21.07. HR-Mass (*m*/*z*): 670.86 [M + H]^+^, found: 670.804 [M + H]^+^.^71^

### 5,10,15,20-Tetrakis(4-methoxyphenyl)porphyrin (4c)

66% yield; FT-IR (KBr, *ν*, cm^−1^): 3433, 3001, 2833, 1689, 1609, 1583, 1462, 1248. ^13^C NMR (126 MHz, DMSO-*d*_6_) *δ* 157.99, 136.64, 133.35, 129.52, 113.65, 105.96, 55.46. HR-Mass (*m*/*z*): 734.86 [M + H]^+^, found: 734.593 [M + H]^+^.^71^

### 5,10,15,20-Tetrakis(4-chlorophenyl)porphyrin (4d)

88% yield; FT-IR (KBr, *ν*, cm^−1^): 3438, 3096, 2882, 1665, 1570, 1468, 845. ^1^H NMR (400 MHz, DMSO-*d*_6_) *δ* 7.96 (s, 8H), 7.29 (d, *J* = 8.0 Hz, 8H), 7.06 (d, *J* = 8.0 Hz, 8H), −2.87 (s, 2H). ^1^H NMR (400 MHz, DMSO-*d*_6_ in D2O) *δ* 8.90 (s, 4H), 7.58 (d, *J* = 7.9 Hz, 4H), 7.34 (d, *J* = 8.0 Hz, 4H). ^13^C NMR (126 MHz, DMSO-*d*_6_) *δ* 143.44, 132.73, 131.01, 130.35, 128.25, 106.37. HR-Mass (*m*/*z*): 752.52 [M + H]^+^, found: 752.512 [M + H]^+^.^71^

### 5,10,15,20-Tetrakis(2,4-dichlorophenyl)porphyrin (4e)

92% yield; FT-IR (KBr, *ν*, cm^−1^): 3436, 3100, 2895, 1695, 1586, 1468, 782, 858. ^1^H NMR (400 MHz, DMSO-*d*_6_) *δ* 8.11 (s, 8H), 7.49 (s, 4H), 7.31 (d, *J* = 8.3, 4H), 6.92 (d, *J* = 8.0, 4H), −2.84 (s, 2H). ^13^C NMR (126 MHz, DMSO-*d*_6_) *δ* 140.88, 134.21, 131.81, 131.46, 128.85, 127.70, 127.32, 106.79. HR-Mass (*m*/*z*): 890.29 [M + H]^+^, found: 890.151 [M + H]^+^.^71^

### 5,10,15,20-Tetra(thiophen-2-yl)porphyrin (4f)

56% yield, FT-IR (KBr, *ν*, cm^−1^): 3403, 3248, 3060, 2895, 1684, 1514, 1415. ^1^H NMR (400 MHz, DMSO-*d*_6_) *δ* 8.21 (s, 8H), 8.09 (d, *J* = 8.2 Hz, 4H), 7.72 (d, *J* = 6.7 Hz, 4H), 7.32 (t, *J* = 8.2 Hz, 4H), −2.78 (s, 2H). HR-Mass (*m*/*z*): 638.84 [M + H]^+^, found: 639.060 [M + H]^+^.

### 5,10,15,20-Tetra(pyridin-2-yl)porphyrin (3g)

82% yield; FT-IR (KBr, *ν*, cm^−1^): 3307, 3099, 2924, 2853, 1691, 1578, 1451. ^1^H NMR (400 MHz, DMSO-*d*_6_) *δ* 8.63 (s, 4H), 8.43 (d, *J* = 7.9 Hz, 4H), 7.95 (d, *J* = 7.9 Hz, 4H), 7.67 (t, *J* = 7.0 Hz, 4H), 7.34 (t, *J* = 7.9 Hz, 4H), −2.81 (s, 2H).

### 5,10,15,20-Tetrayltetrakis(benzene-4,1-diyl) tetrakis(4-methylbenzenesulfonate) (4h)

63% yield; FT-IR (KBr, *ν*, cm^−1^): 3401, 3060, 2920, 2859, 1685, 1596, 1498, 1371, 1152. ^1^H NMR (500 MHz, DMSO-*d*_6_) *δ* 7.99 (s, 8H), 7.71 (d, *J* = 8.9 Hz, 8H), 7.44 (d, *J* = 7.9 Hz, 8H), 7.08 (d, *J* = 9.0 Hz, 8H), 6.90 (d, *J* = 9.2 Hz, 8H), 2.40 (s, 12H), −2.79 (s, 2H). ^13^C NMR (75 MHz, DMSO) *δ* 147.69, 146.14, 133.14, 132.70, 132.10, 130.62, 129.91, 128.58, 121.96, 117.44, 21.62. HR-Mass (*m*/*z*): 1295.48 [M + H]^+^, found: 1295.453 [M + H]^+^.

## Data availability

Data will be made available on request.

## Author contributions

Azin Kharazmi: involved in conceptualization, methodology, resources, writing—original draft, reviewing and editing, formal analysis. Ramin Ghorbani-Vaghei: took part in conceptualization, visualization, investigation, supervision, reviewing and editing. Ardeshir Khazaei: supervision. Idris Karakaya: took part in data curation, formal analysis, reviewing and editing. Rahman Karimi-Nami: reviewing and editing, involved in data curation and formal analysis.

## Conflicts of interest

The authors declare that they have no known competing fnancial interests or personal relationships that could have appeared to influence the work reported in this paper.

## Supplementary Material

RA-015-D4RA07875F-s001
