# Supplementary material for: Application of novel silica stabilized on a covalent triazine framework as a highly efficient heterogeneous and recyclable catalyst in the effective green synthesis of porphyrins
Source: RSC Adv. 2025 Jan 13;15(2):1081–94. doi: 10.1039/d4ra07875f (PMC11726313; doi:10.1039/d4ra07875f)
Supplement: RA-015-D4RA07875F-s001 [file RA-015-D4RA07875F-s001.pdf]

Supplementary Information (SI) for RSC Advances.
This journal is © The Royal Society of Chemistry 2025

Supporting Information

Application of novel silica stabilized on covalent triazine framework as a highly efficient heterogeneous and recyclable catalyst in the effective green synthesis of porphyrins

Azin Kharazmi,[a] Ramin Ghorbani-Vaghei,*[,a,b] Ardeshir Khazaei,[a] Idris Karakaya,[c] and Rahman Karimi-Nami[d]

[a]Department of Organic Chemistry, Faculty of Chemistry and Petroleum Sciences, Bu-Ali Sina University, Hamedan 6517838683, Iran.

[b]Department of Organic Chemistry, Faculty of Chemistry, University of Guilan, Rasht, Iran

[c]Department of Chemistry, College of Basic Sciences, Gebze Technical University, 41400 Gebze, Türkiye.

[d]Department of Chemistry, Faculty of Science, University of Maragheh, P.O Box 55181-83111, Maragheh, Iran.

**Corresponding author: Phone: +989183122123; Fax: +988138380709; E-mail address: rgvaghei@yahoo.com & ghorbani@basu.ac.ir*

Table of contents

Section	***Page number***
Copies of FT-IR, NMR and HR-Mass spectra of products	*S3-S20*

S1. Copies of NMR spectra of aldehyde, amine, TPT-TAT/silica (entries 1-3)

S1-1. 2,4,6-tris(4-formylphenoxy)-1,3,5-triazine (1a)

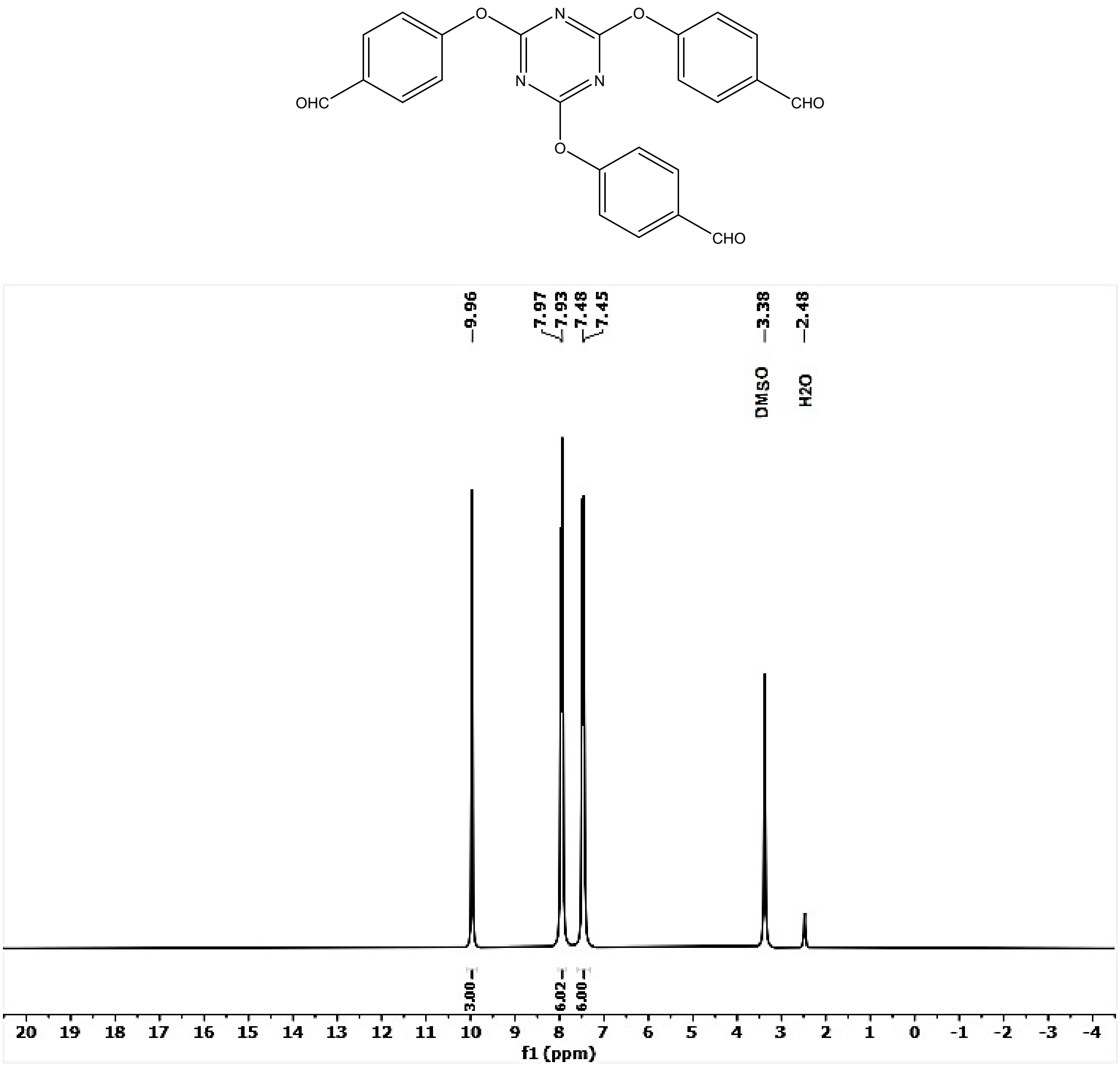

Figure S1. [1] H NMR spectra of 2,4,6-tris(4-formylphenoxy)-1,3,5-triazine (1a).

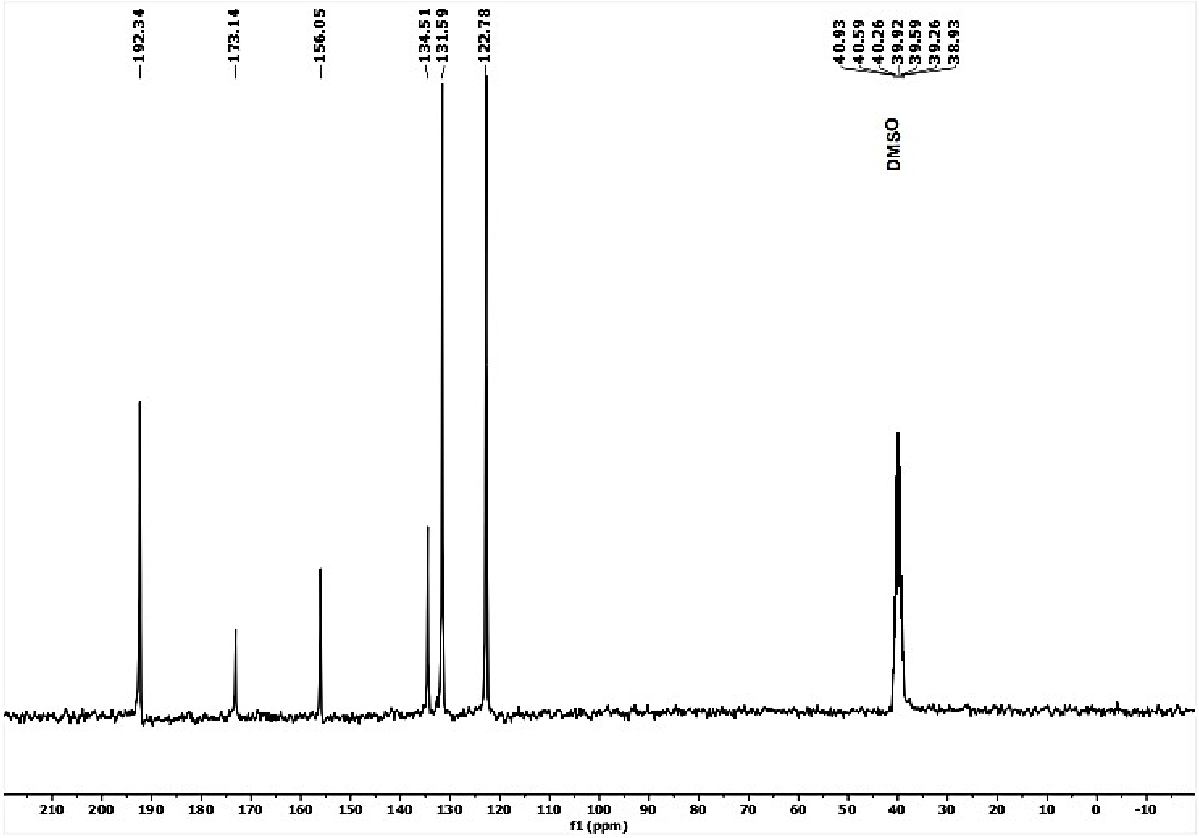

Figure S2. [13] CNMR spectra of 2,4,6-tris(4-formylphenoxy)-1,3,5-triazine (1a).

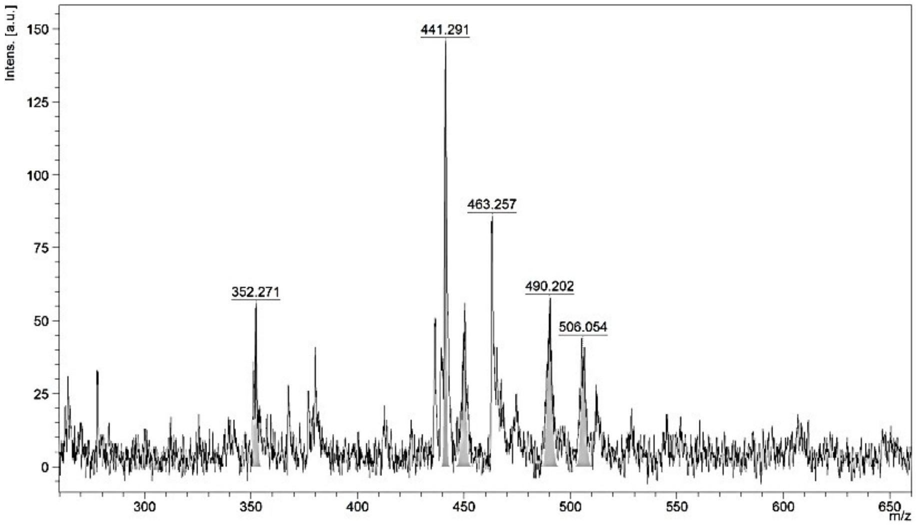

m/z	S/N	Quality Fac.	Res.	Intens.	Area
352.271	5		233	56.0	157
436.433				51.0	
441.291	12		364	146	354
449.898	4		189	46.0	160
463.257				86.0	
490.202	5		190	58.0	206
506.054	3		188	38.0	140

Figure S3. HR-Mass spectra of 2,4,6-tris(4-formylphenoxy)-1,3,5-triazine (1a).

S1-2. ***N*[2], *N*[4], *N*[6]-tris(4-(aminomethyl) benzyl)-1,3,5-triazine-2,4,6-triamine (2a)**

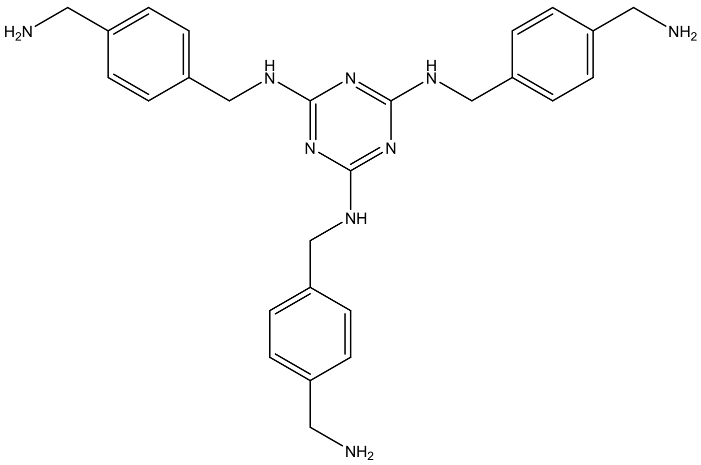

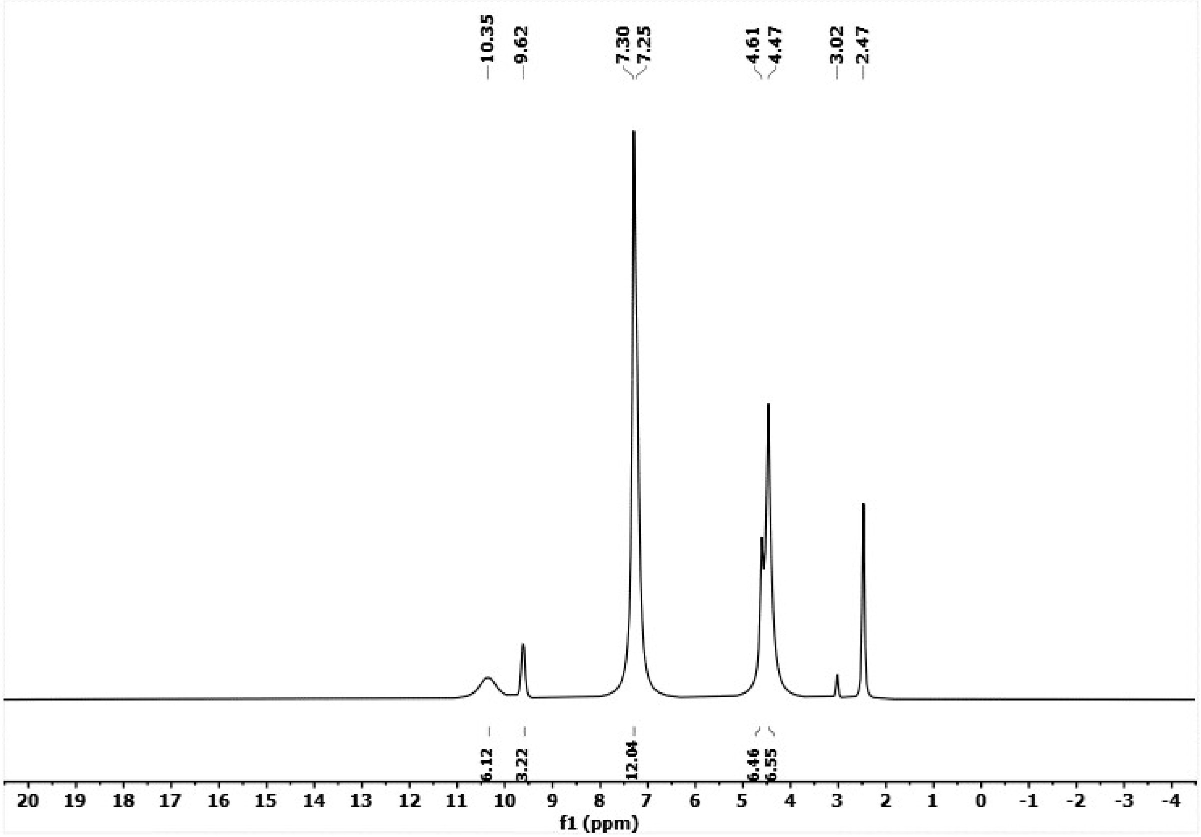

Figure S4. ^{1}H NMR spectra of *N*[2], *N*[4], *N*[6]-tris(4-(aminomethyl) benzyl)-1,3,5-triazine-2,4,6-triamine (2a).

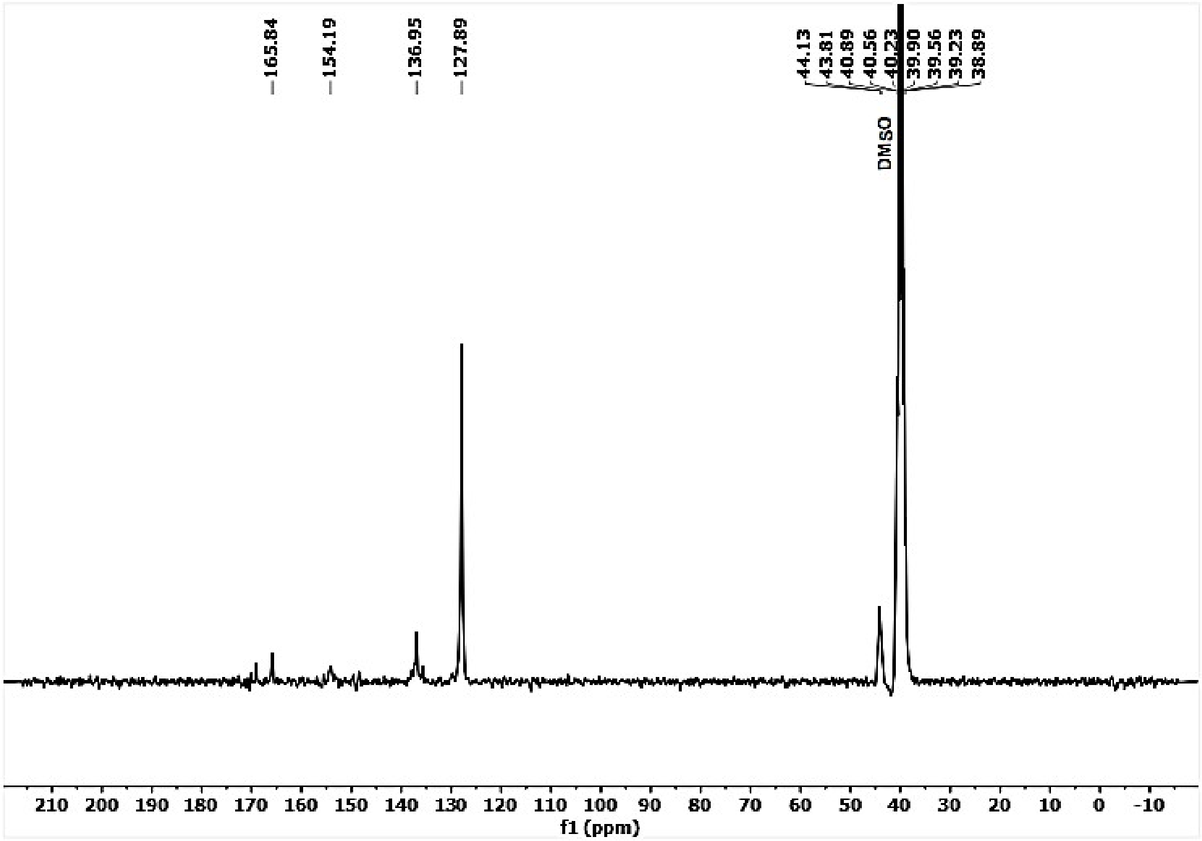

Figure S5. [13] CNMR spectra of N^2, N^4, N^6-tris(4-(aminomethyl) benzyl)-1,3,5-triazine-2,4,6-triamine (2a).

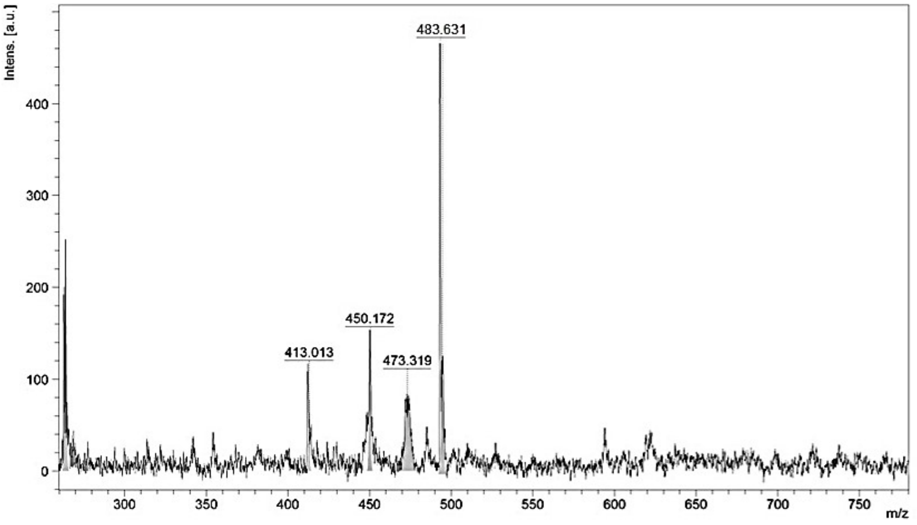

m/z	S/N	Quality Fac.	Res.	Intens.	Area
263.868	15		188	252	426
413.013	3		379	57.0	245
450.172	8		357	154	480
473.319	4		114	78.0	381
483.621	7		196	123	570

Figure S6. HR-Mass spectra of N^2, N^4, N^6-tris(4-(aminomethyl) benzyl)-1,3,5-triazine-2,4,6-triamine (2a).

S1-3. TPT/TAT (3a)

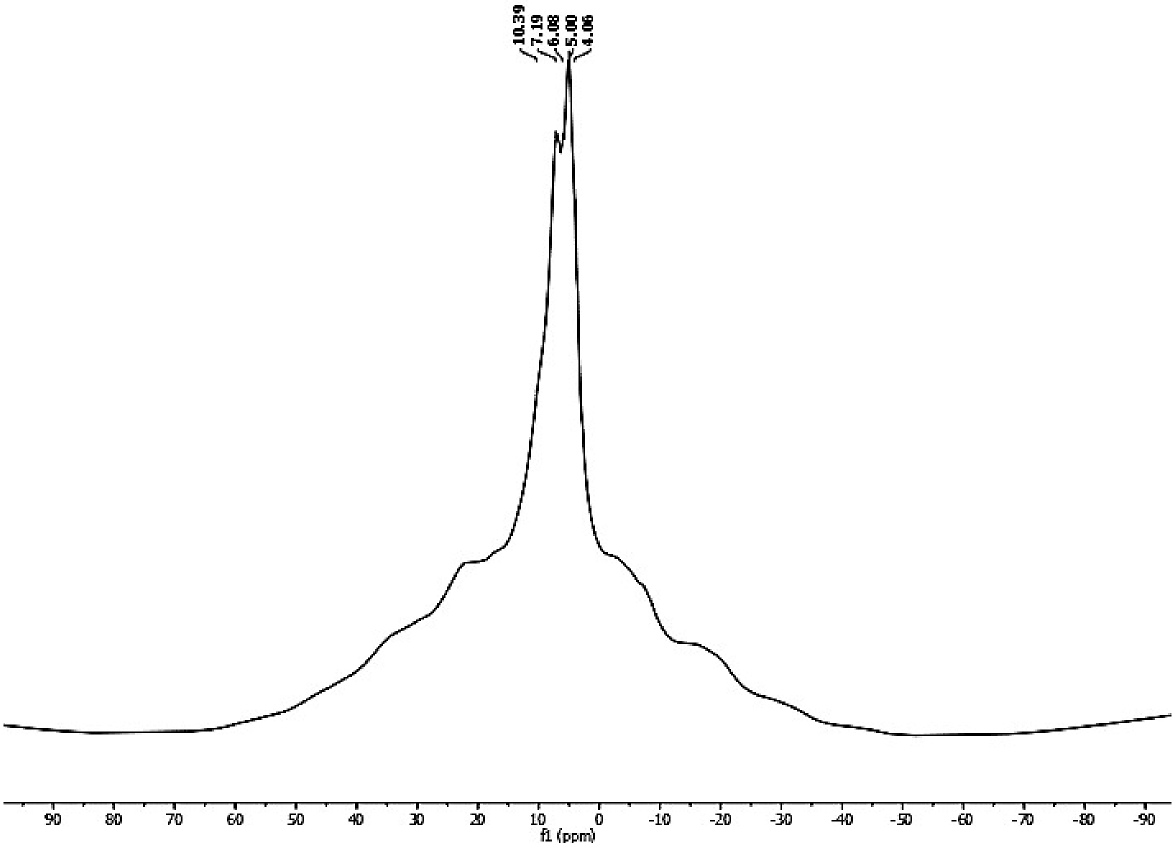

Figure S7. ^{1}H-SSNMR spectra of covalent triazine framework (TPT-TAT) (3a).

S2. Copies of FT-IR, NMR and HR-Mass spectra of products (entries 1-8)

S2-1. 5,10,15,20-Tetraphenylporphyrin (4a)

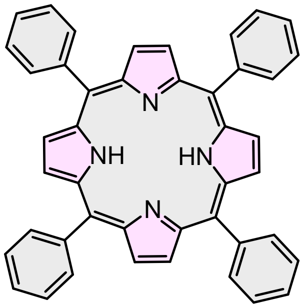

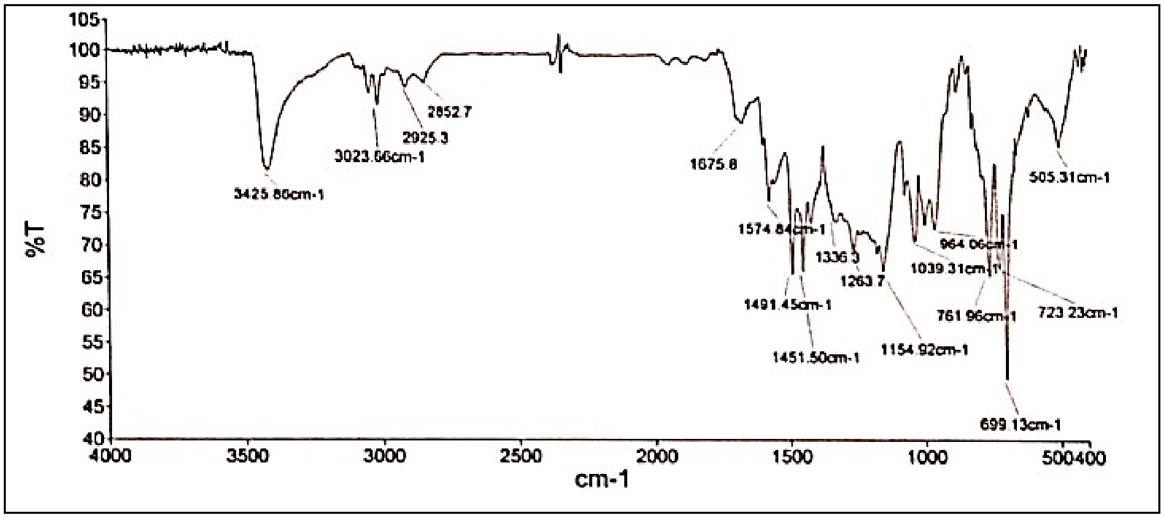

Figure S8. FT-IR spectra of 5,10,15,20-Tetraphenylporphyrin (4a).

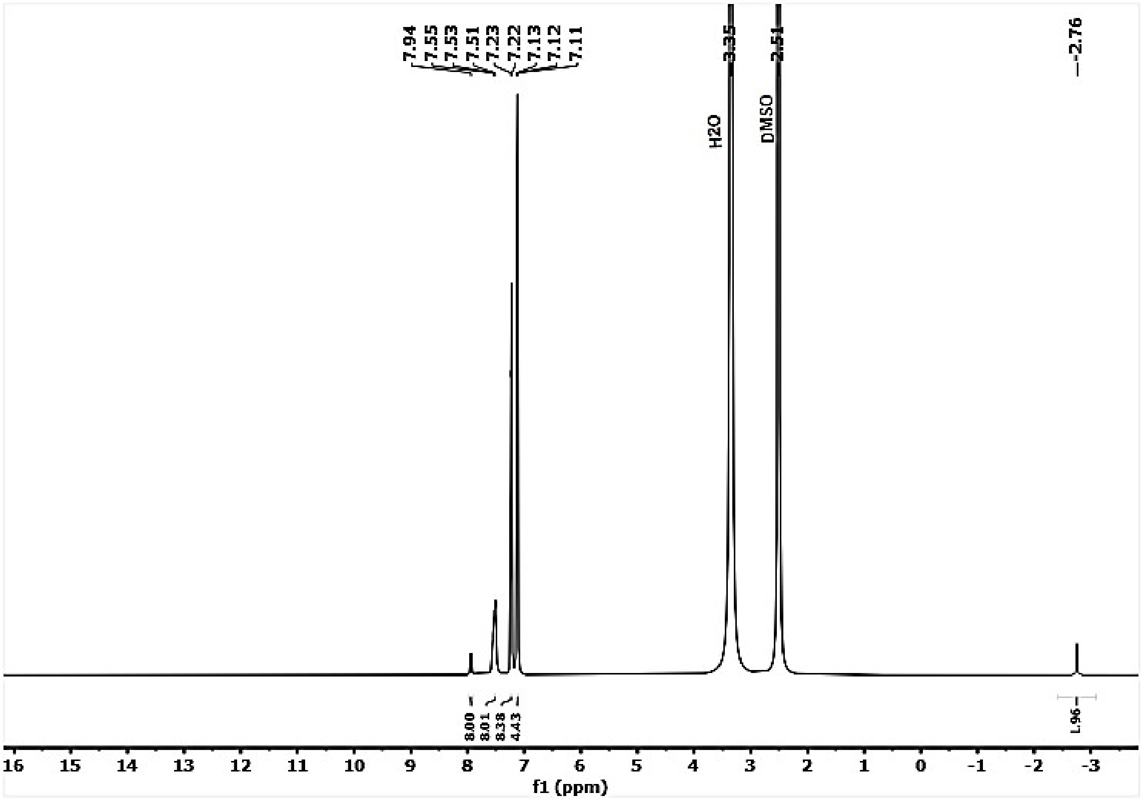

Figure S9. [1] H NMR spectra of 5,10,15,20-Tetraphenylporphyrin (4a).

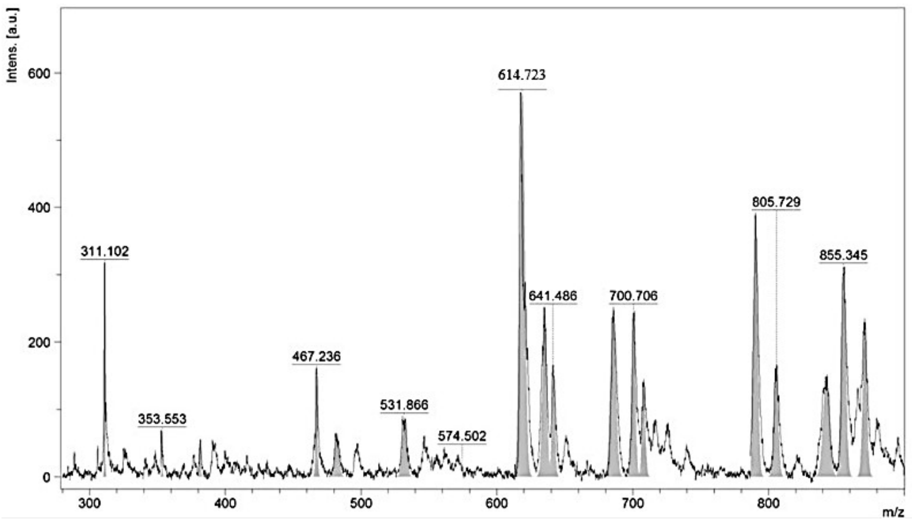

m/z	S/N	Quality Fac.	Res.	Intens.	Area
311.102	17		421	318	603
353.553	4		414	69.0	129
381.460	3		228	55.0	123
467.236	8		268	152	502
482.247	3		125	65.0	297
531.866	4		134	90.0	442
574.502				19.0	
614.723	22		135	571	2620
635.081	10		163	252	1241
641.486	6		225	164	566
685.826	8		146	244	1283
700.706	8		210	246	1046
708.152	4		237	138	667
790.630	9		209	371	1812
805.729	4		211	168	813
842.604	3		147	149	1048
855.345	7		210	313	1641
870.854	5		230	222	1689

Figure S10. HR-Mass spectra of 5,10,15,20-Tetraphenylporphyrin (4a).

S2-2. 5,10,15,20-Tetrakis(4-methylphenyl)porphyrin (4b)

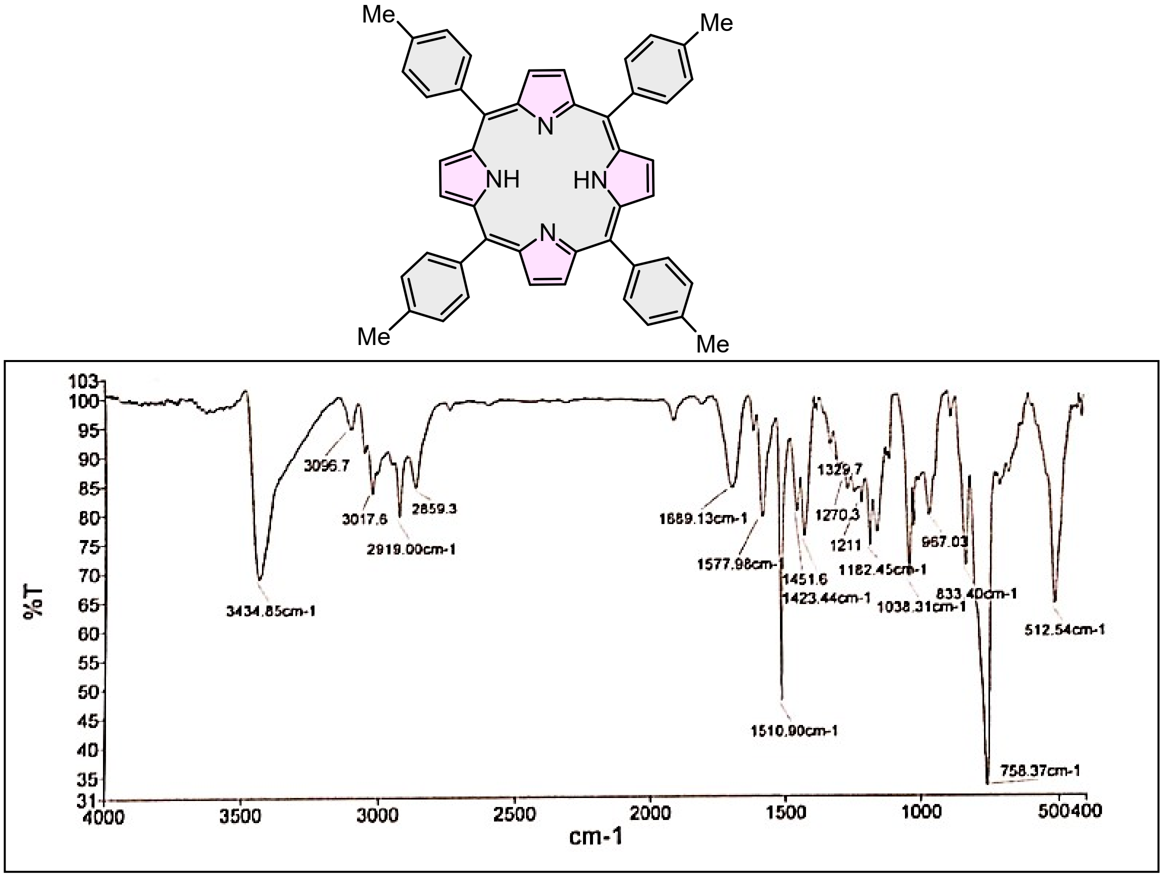

Figure S11. FT-IR spectra of 5,10,15,20-Tetrakis(4-methylphenyl)porphyrin (4b).

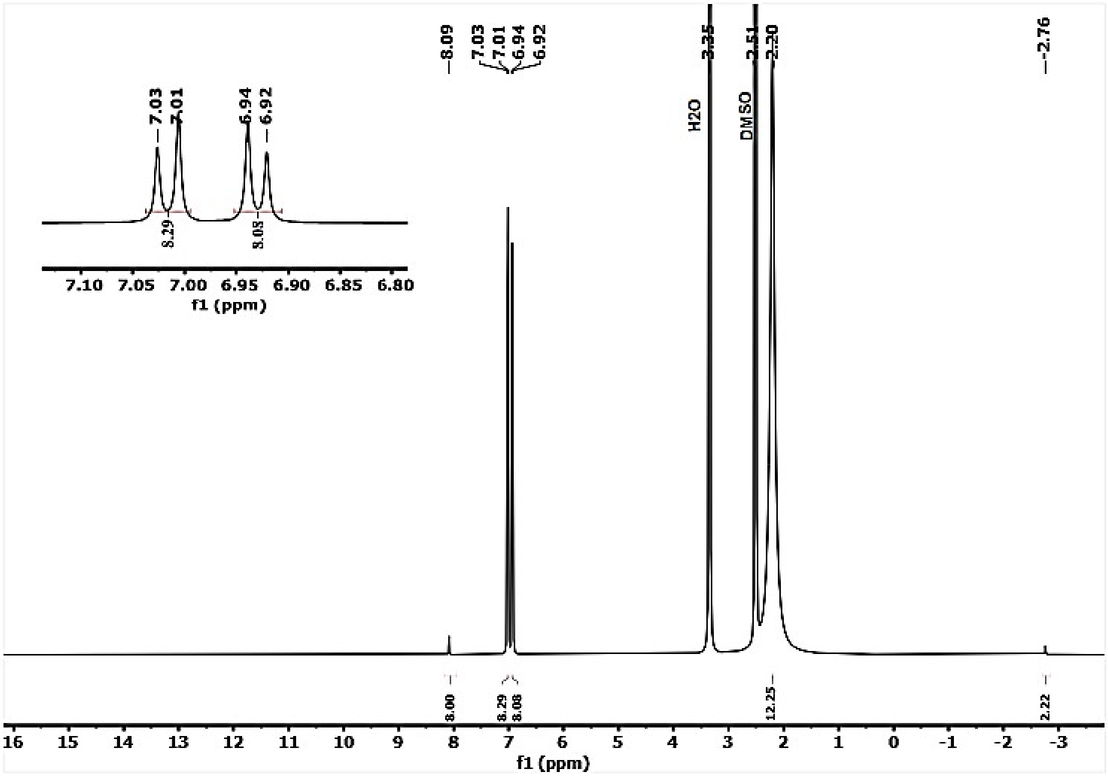

Figure S12. [1] H NMR spectra of 5,10,15,20-Tetrakis(4-methylphenyl)porphyrin (4b).

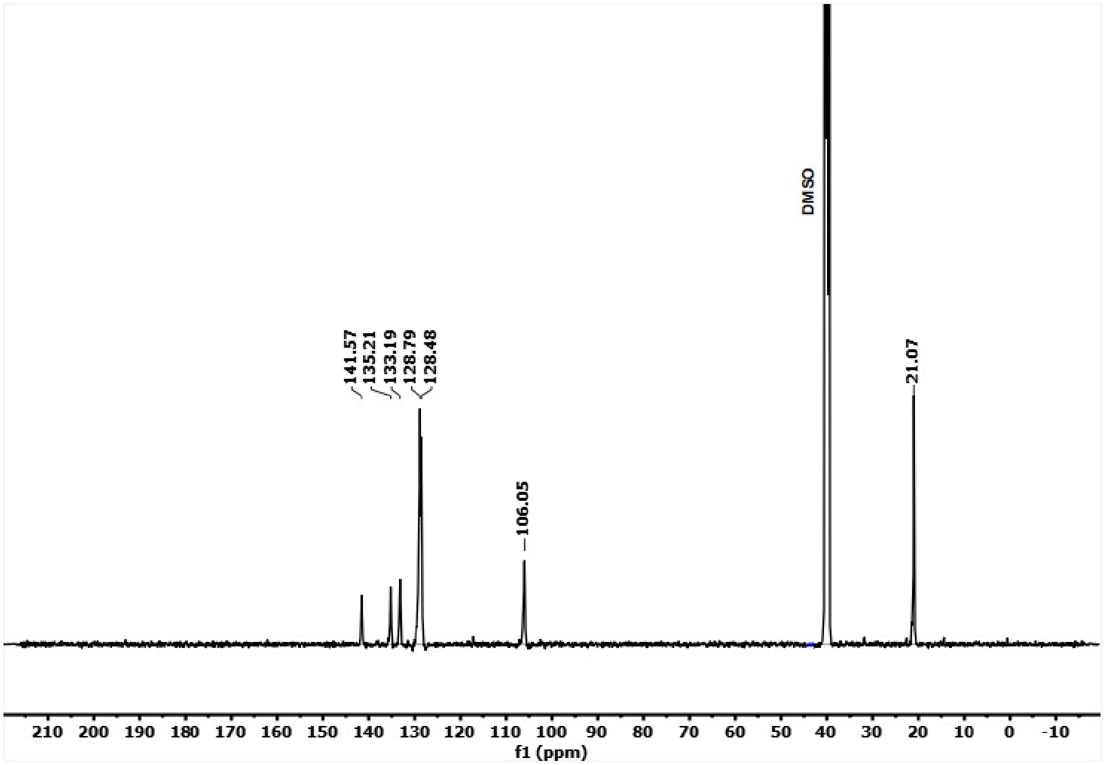

Figure S13. [13] CNMR spectra of 5,10,15,20-Tetrakis(4-methylphenyl)porphyrin (4b).

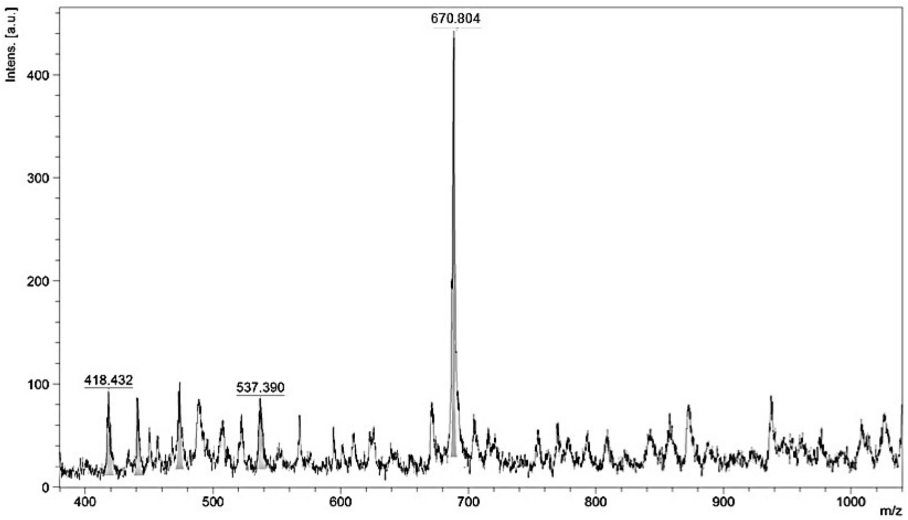

m/z	S/N	Quality Fac.	Res.	Intens.	Area
418.432	5		131	93.0	237
441.267	4		175	87.1	179
473.995	5		185	102	330
537.390	4		130	86.0	235
670.804	22		265	425	1202

Figure S14. HR-Mass spectra of 5,10,15,20-Tetrakis(4-methylphenyl)porphyrin (4b).

S2-3. 5,10,15,20-Tetrakis(4-methoxyphenyl)porphyrin (4c)

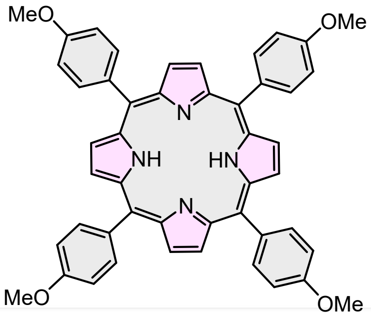

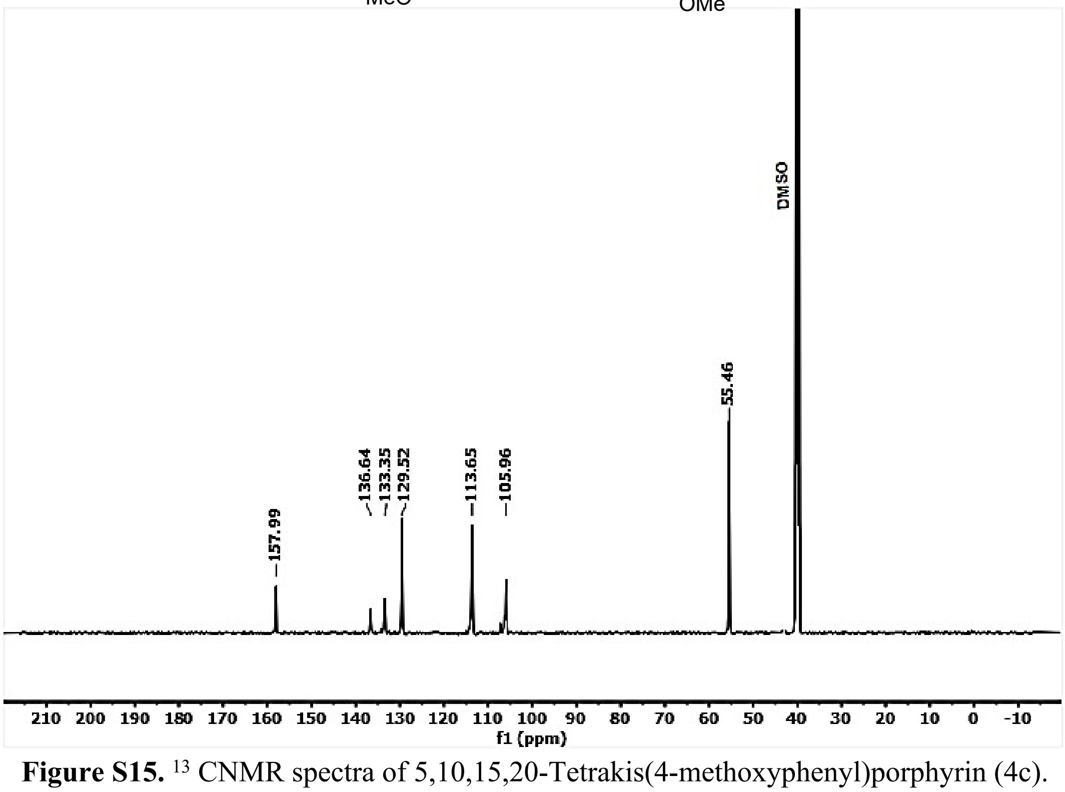

Figure S15. [13] CNMR spectra of 5,10,15,20-Tetrakis(4-methoxyphenyl)porphyrin (4c).

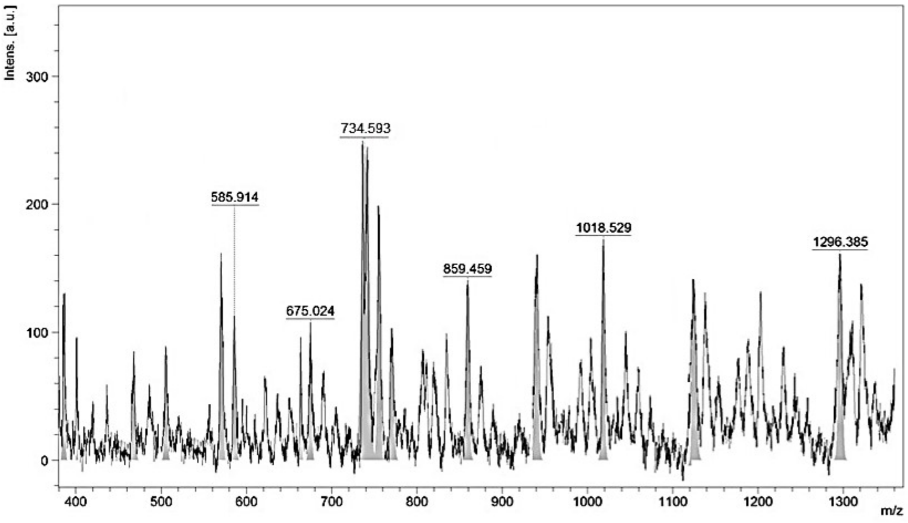

m/z	S/N	Quality Fac.	Res.	Intens.	Area
385.990	4		133	105	467
401.300	4		357	96.0	231
467.509	3		114	85.0	321
505.714	3		147	88.0	399
570.514	6		140	150	662
585.914	4		155	105	422
664.137	3		360	96.0	239
675.024	4		195	108	455
734.593	8		92.0	250	1764
740.676	8		92.1	245	1764
755.406	6		175	199	1092
770.551	3		152	100	645
859.459	4		189	141	738
939.990	4		185	147	1012
1018.529	4		254	173	805
1124.522	3		177	142	1085
1296.385	4		245	162	1137

Figure S16. HR-Mass spectra of 5,10,15,20-Tetrakis(4-methoxyphenyl)porphyrin (4c).

S2-4. 5,10,15,20-Tetrakis(4-chlorophenyl)porphyrin (4d)

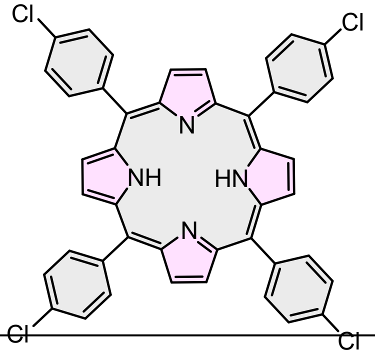

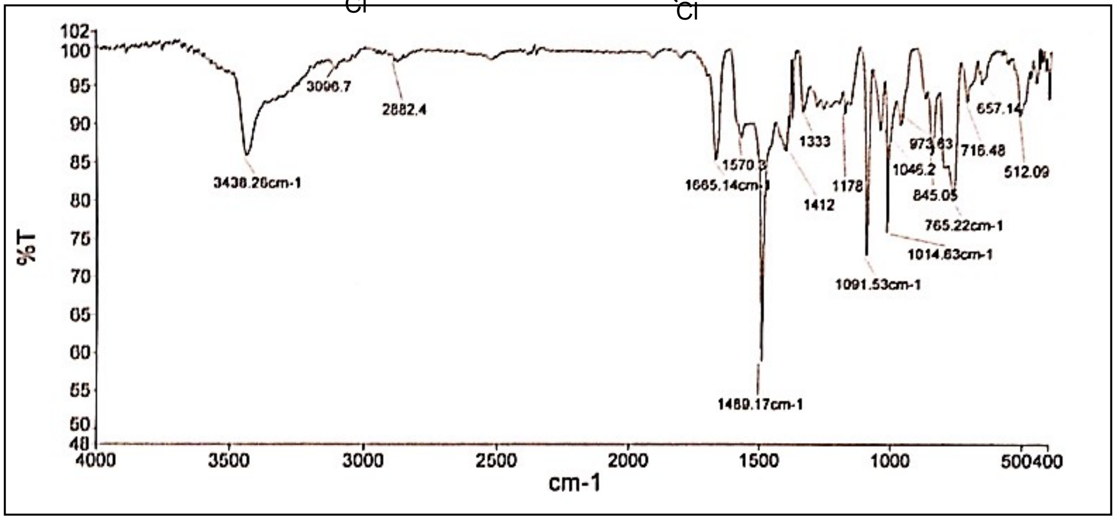

Figure S17. FT-IR spectra of 5,10,15,20-Tetrakis(4-chlorophenyl)porphyrin (4d).

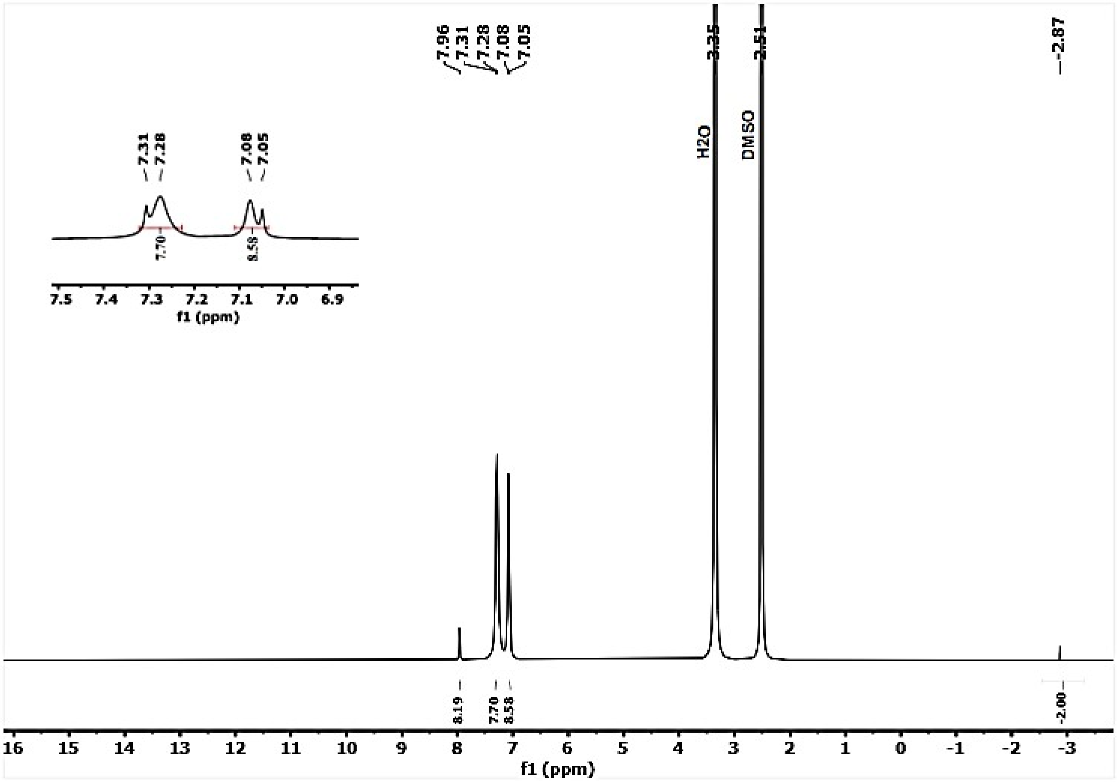

Figure S18. [1] H NMR spectra of 5,10,15,20-Tetrakis(4-chlorophenyl)porphyrin (4d).

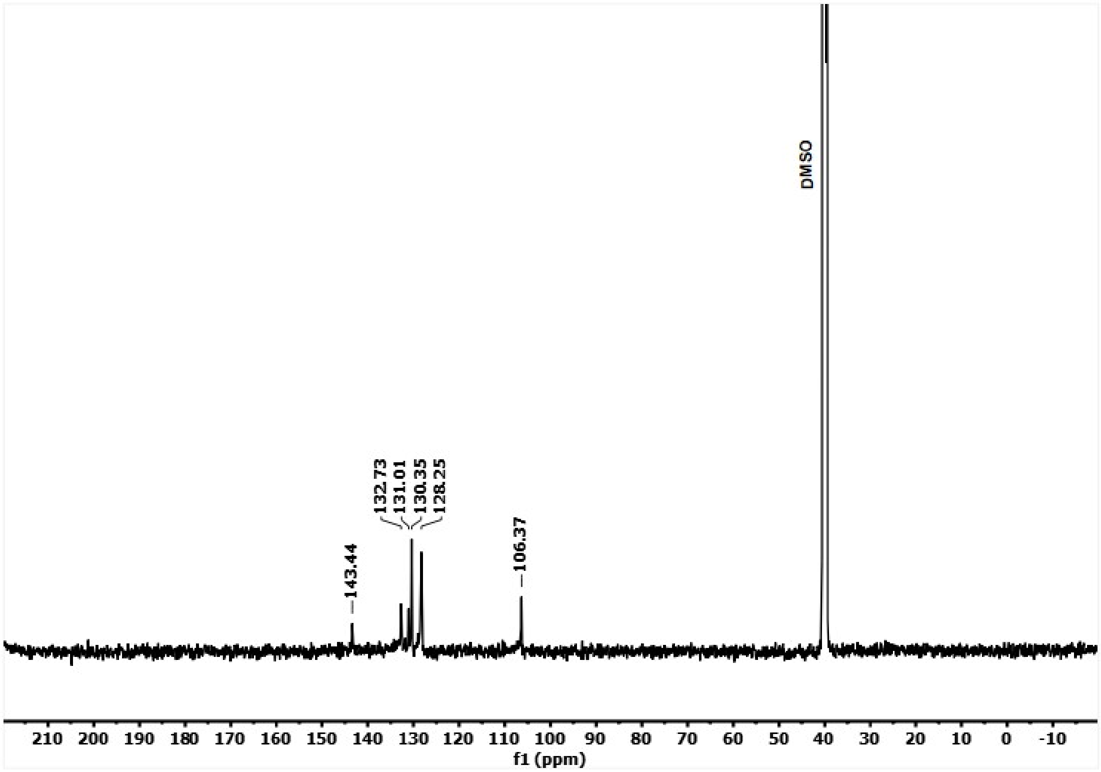

Figure S19. [13] CNMR spectra of 5,10,15,20-Tetrakis(4-chlorophenyl)porphyrin (4d).

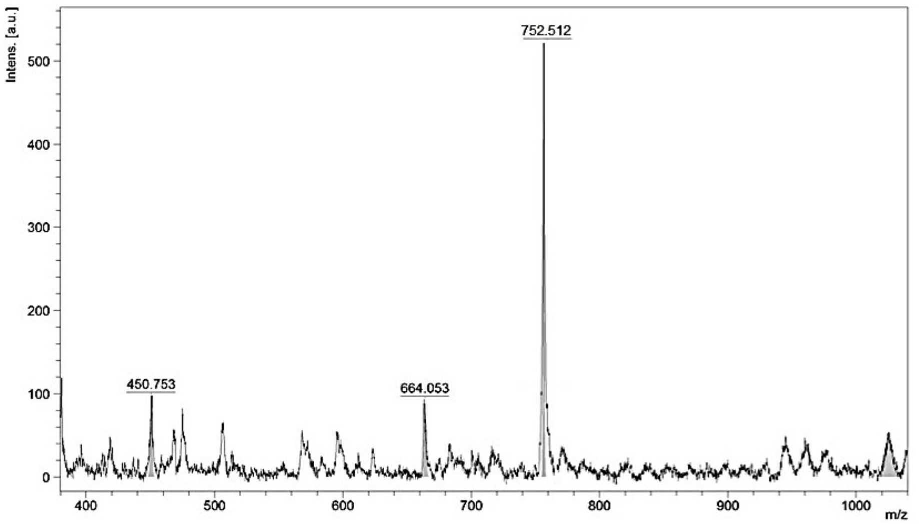

m/z	S/N	Quality Fac.	Res.	Intens.	Area
450.753	5		232	98.0	253
664.053	5		308	93.0	255
752.512	6		182	102	537
1025.266	3		140	54.0	352

Figure S20. HR-Mass spectra of 5,10,15,20-Tetrakis(4-chlorophenyl)porphyrin (4d).

S2-5. 5,10,15,20-Tetrakis(2,4-dichlorophenyl)porphyrin (4e)

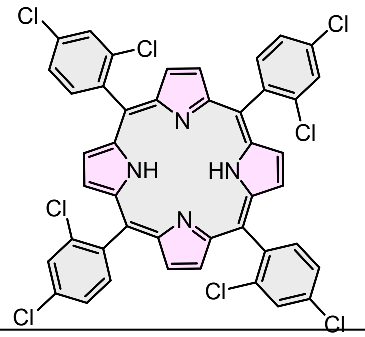

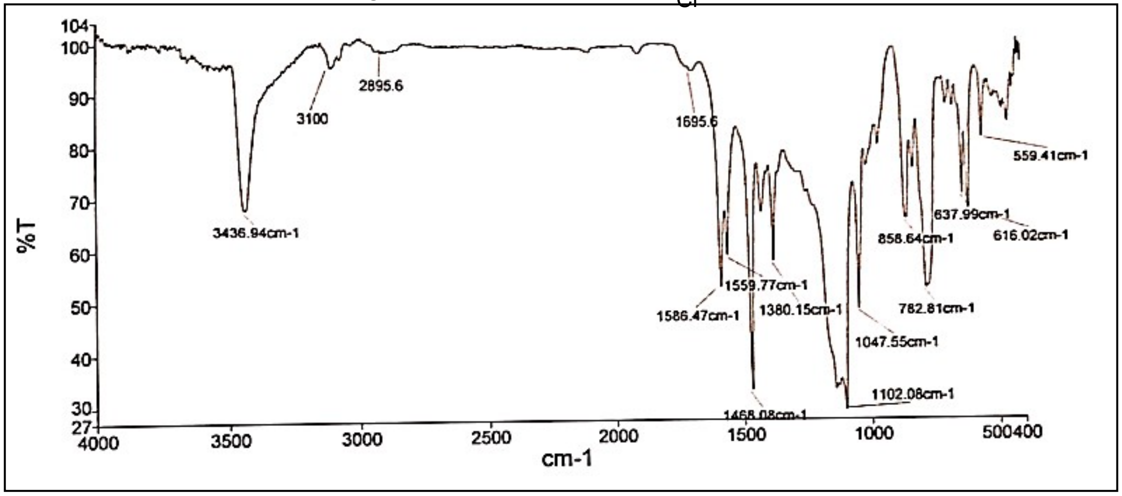

Figure S21. FT-IR spectra of 5,10,15,20-Tetrakis(2,4-dichlorophenyl)porphyrin (4e).

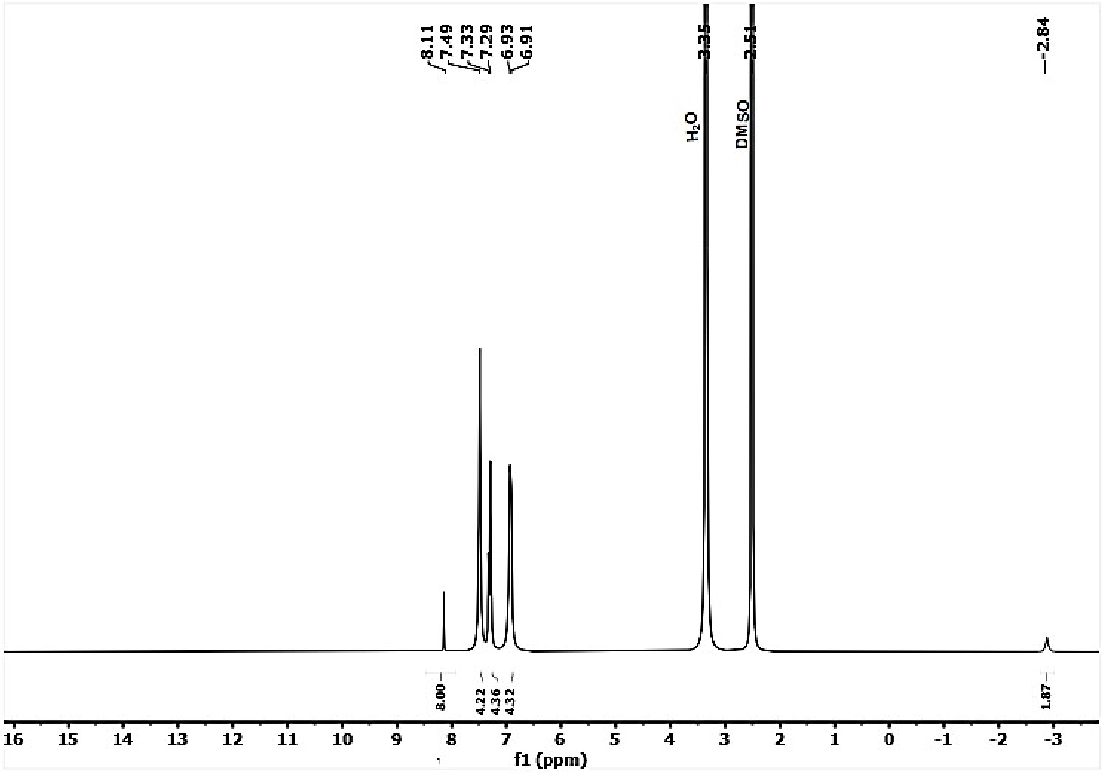

Figure S22. [1] H NMR spectra of 5,10,15,20-Tetrakis(2,4-dichlorophenyl)porphyrin (4e).

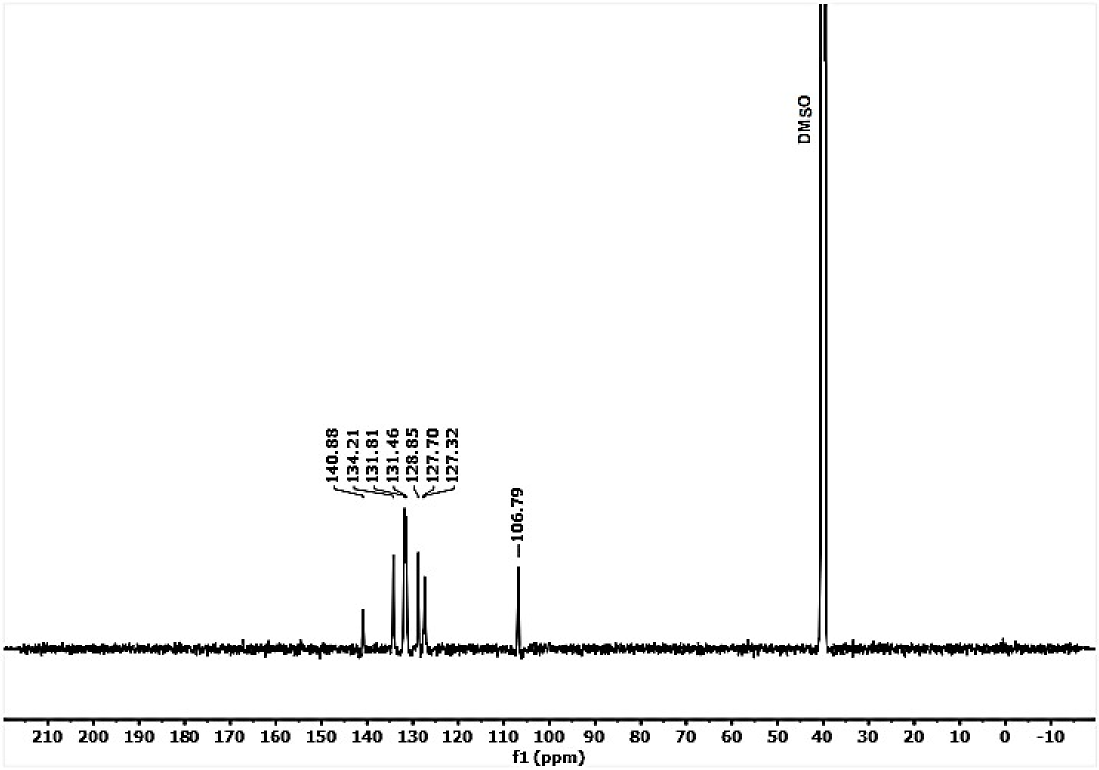

Figure S23. [13] CNMR spectra of 5,10,15,20-Tetrakis(2,4-dichlorophenyl)porphyrin (4e).

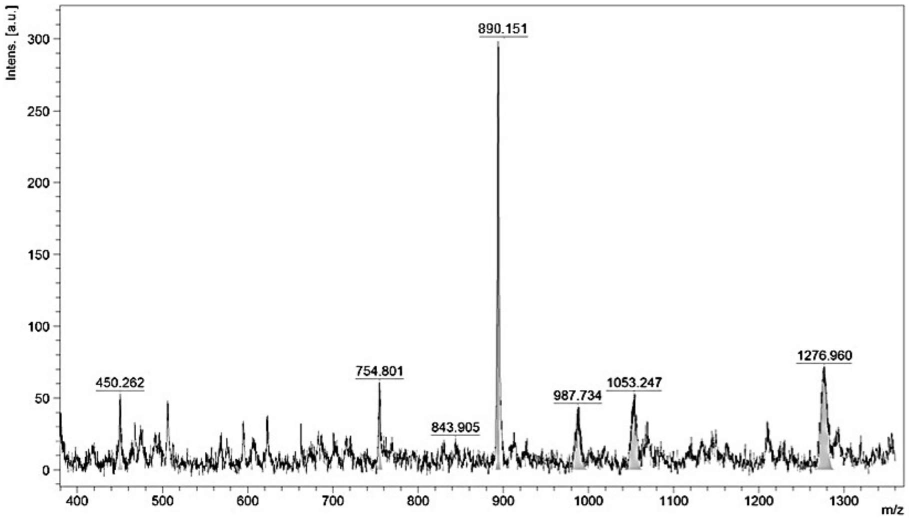

m/z	S/N	Quality Fac.	Res.	Intens.	Area
450.262	4		189	53.0	164
754.801	4		310	61.0	275
843.905				22.0	
890.151	20		242	300	897
987.734	3		124	44.0	277
1053.247	4		147	49.0	380
1276.960	5		165	72.0	643

Figure S24. HR-Mass spectra of 5,10,15,20-Tetrakis(2,4-dichlorophenyl)porphyrin (4e).

S2-6. 5,10,15,20-tetra(thiophen-2-yl)porphyrin (4f)

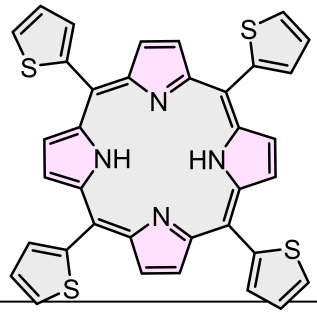

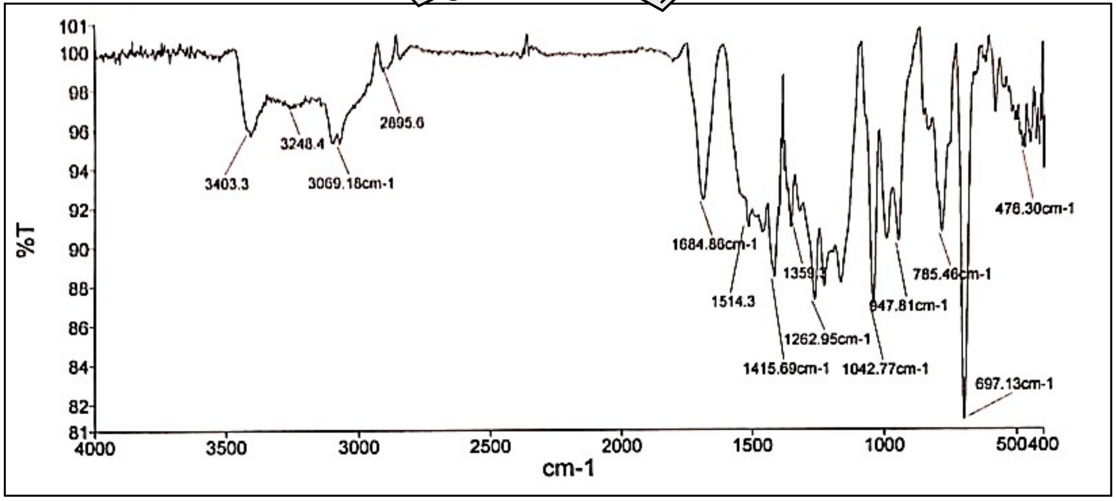

Figure S25. FT-IR spectra of 5,10,15,20-tetra(thiophen-2-yl)porphyrin (4f).

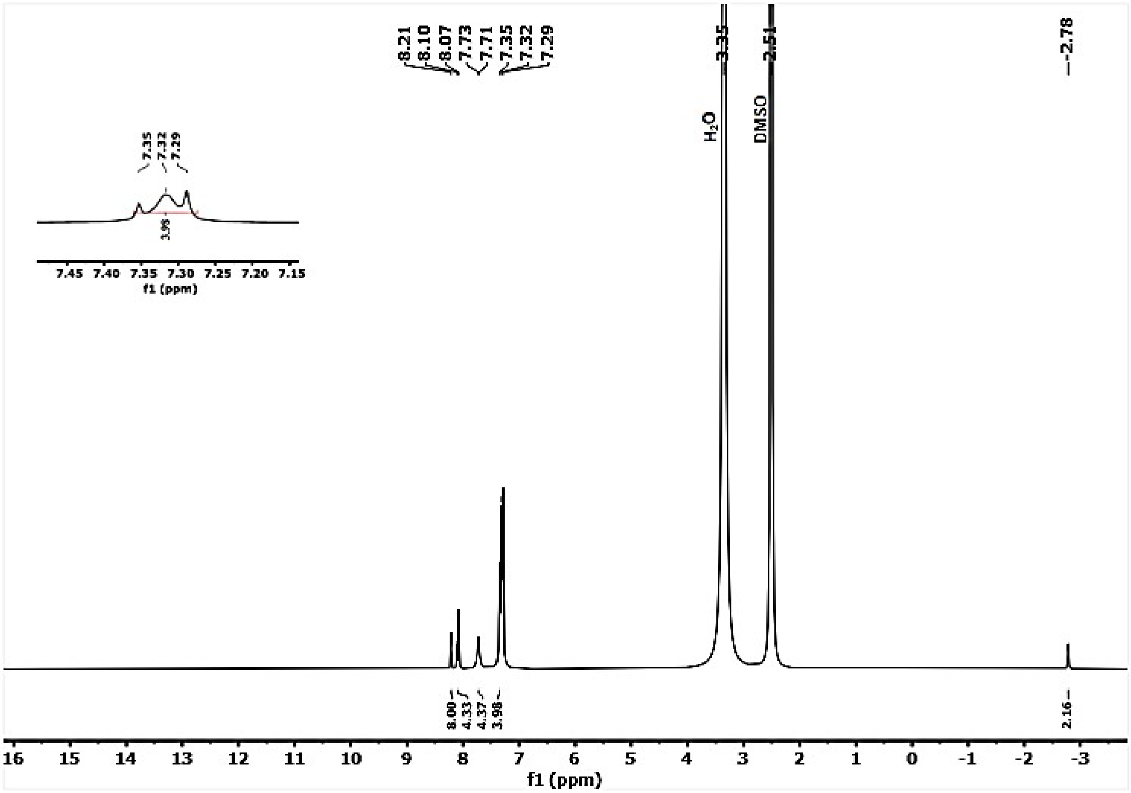

Figure S26. [1] H NMR spectra of 5,10,15,20-tetra(thiophen-2-yl)porphyrin (4f).

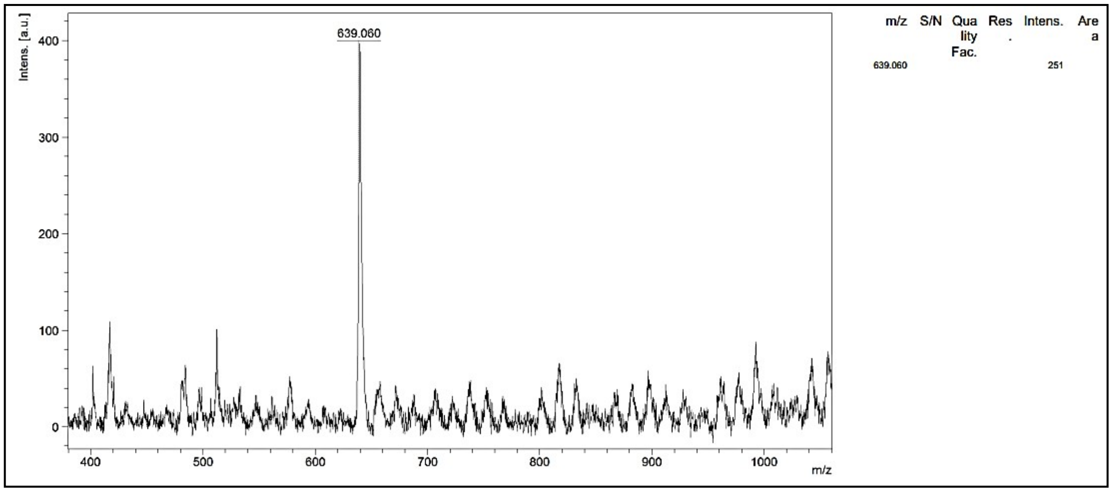

Figure S27. HR-Mass spectra of 5,10,15,20-tetra(thiophen-2-yl)porphyrin (4f).

S2-7. 5,10,15,20-tetra(pyridin-2-yl)porphyrin (3g)

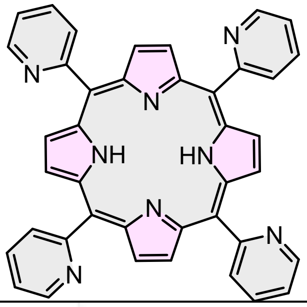

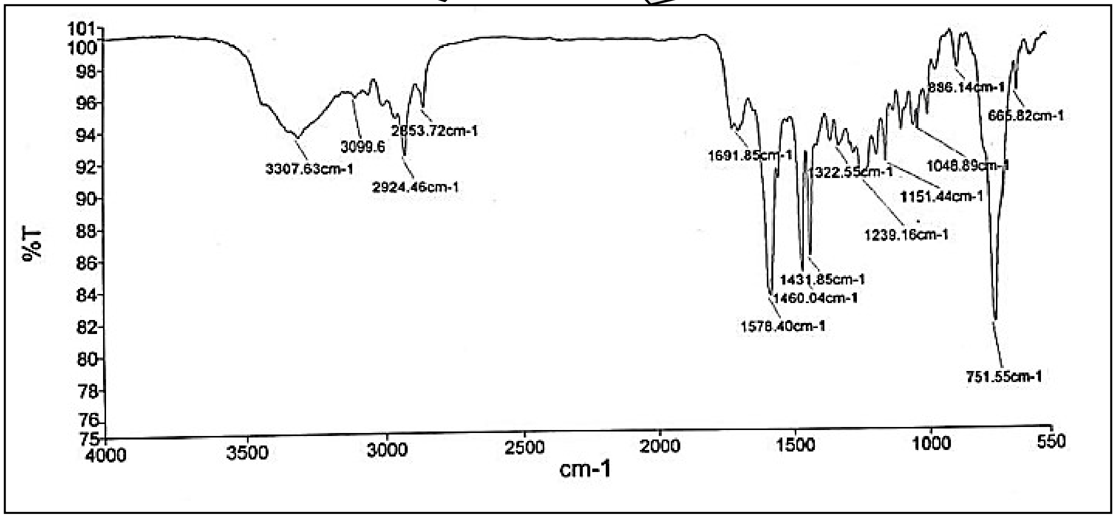

Figure S28. FT-IR spectra of 5,10,15,20-tetra(pyridin-2-yl)porphyrin (4g).

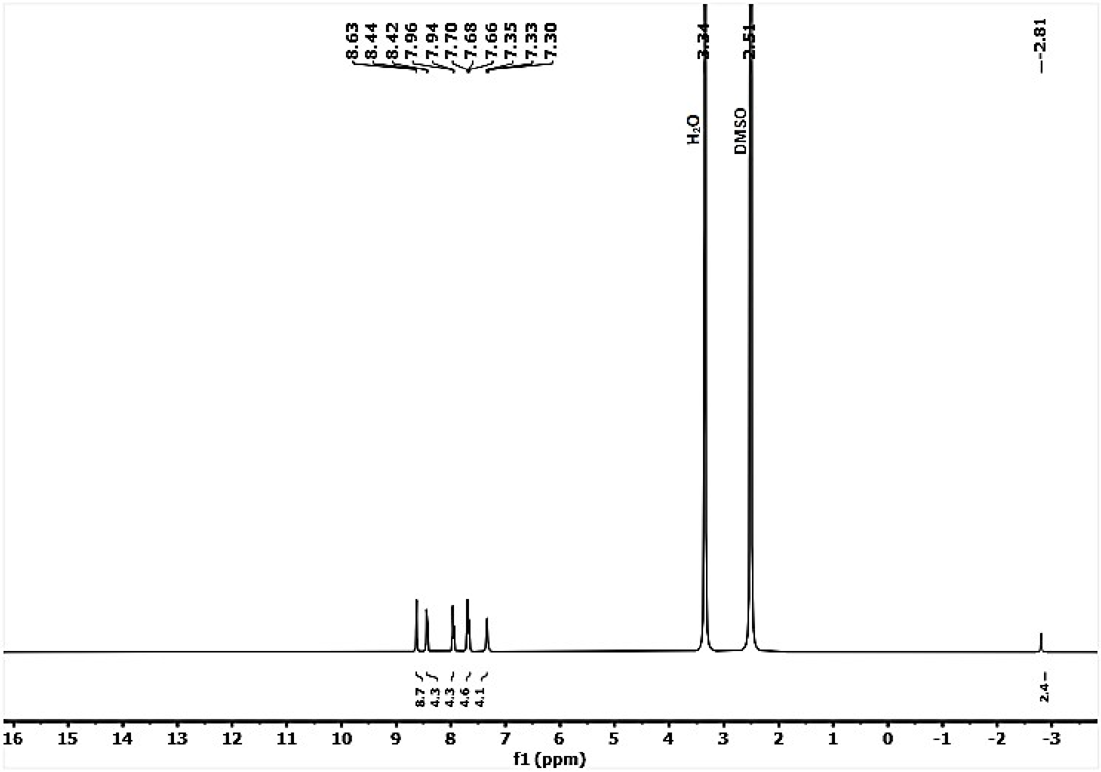

Figure S29. [1] H NMR spectra of 5,10,15,20-tetra(pyridin-2-yl)porphyrin (4g).

S2-8. porphyrin-5,10,15,20-tetrayltetrakis(benzene-4,1-diyl) tetrakis(4-methylbenzenesulfonate) (4h)

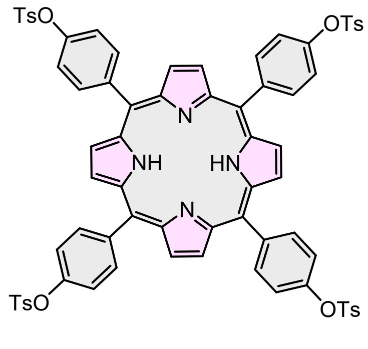

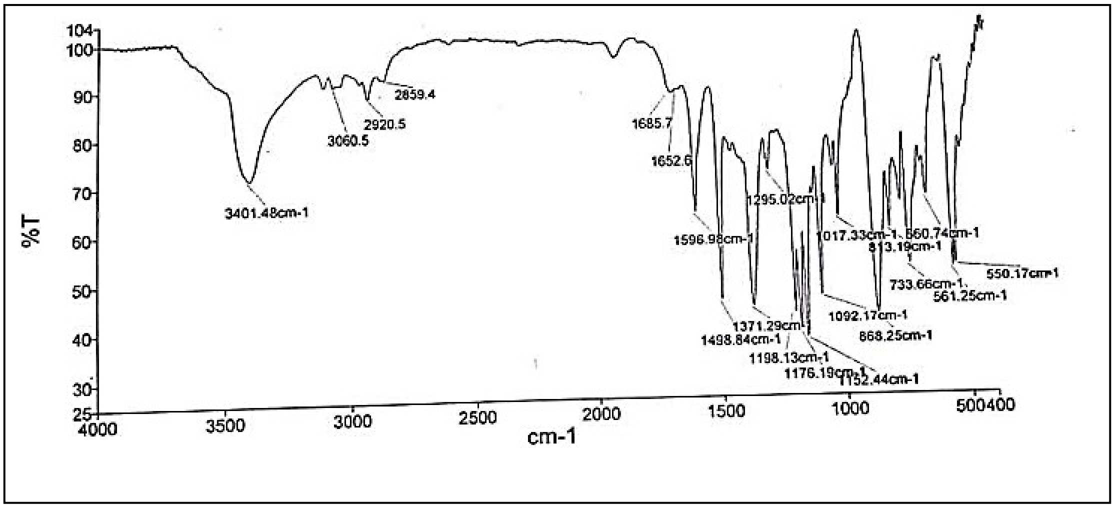

Figure S30. FT-IR spectra of porphyrin-5,10,15,20-tetrayltetrakis(benzene-4,1-diyl) tetrakis(4-methylbenzenesulfonate) (4h).

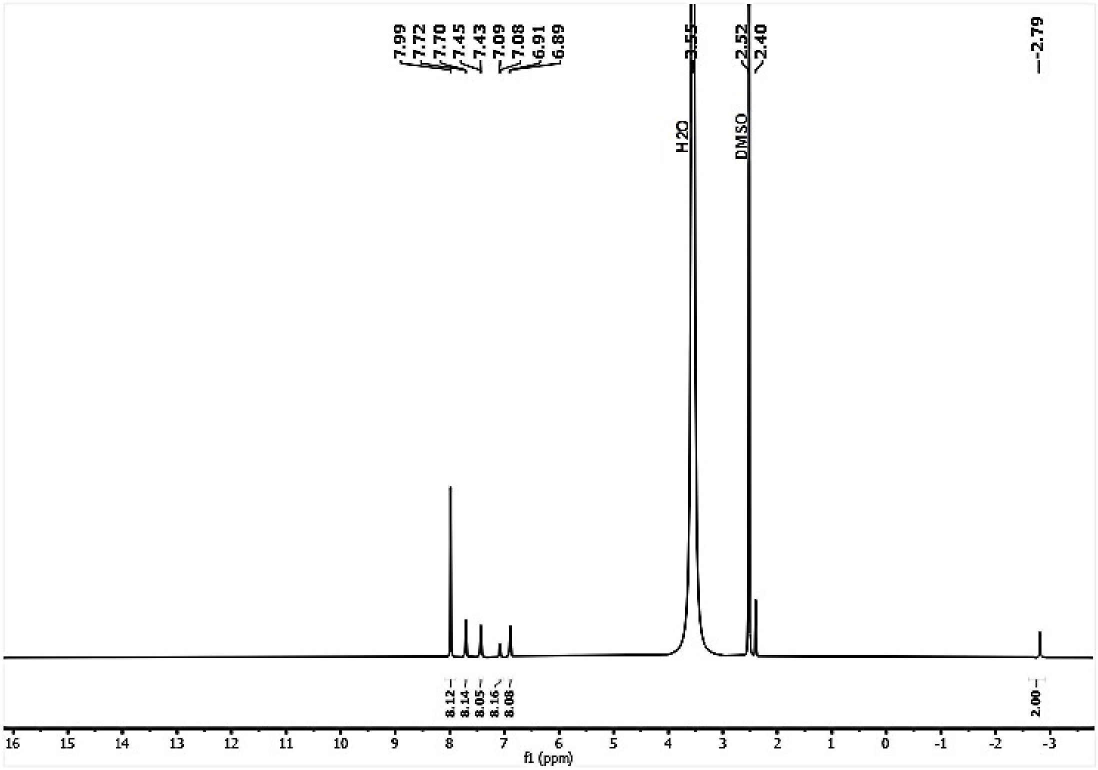

Figure S31. ^{1}H NMR spectra of porphyrin-5,10,15,20-tetrayltetrakis(benzene-4,1-diyl) tetrakis(4-methylbenzenesulfonate) (4h).

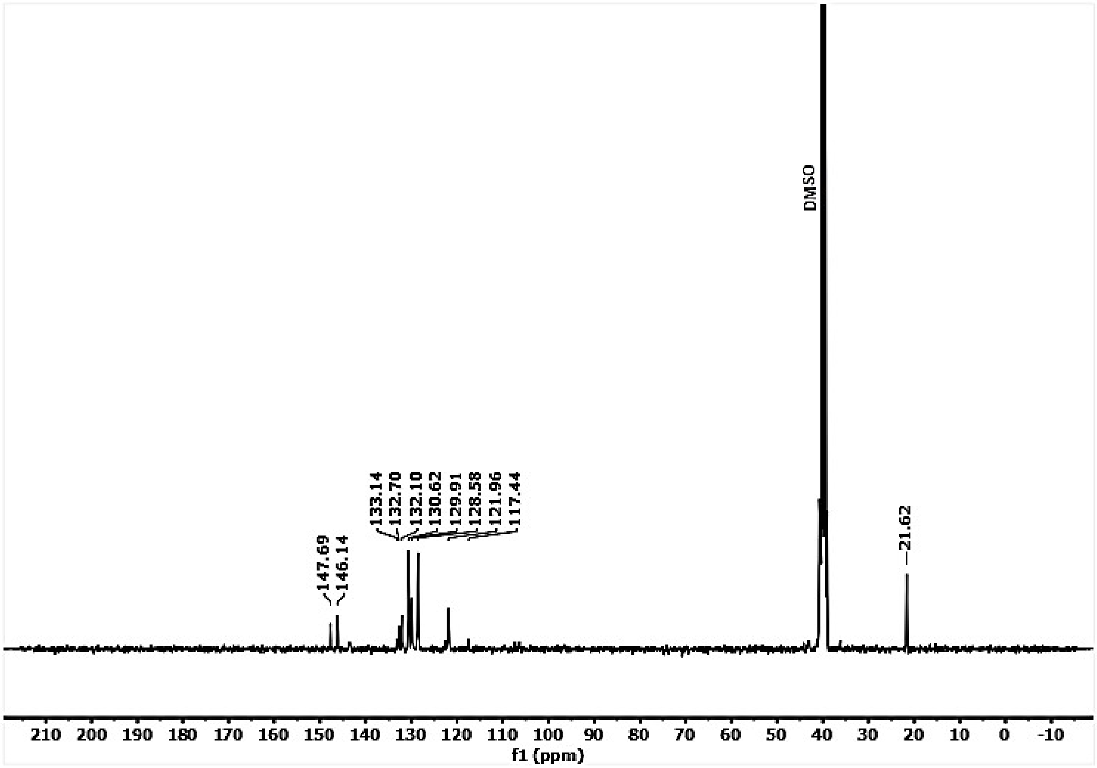

Figure S32. ^{13}CNMR spectra of porphyrin-5,10,15,20-tetrayltetrakis(benzene-4,1-diyl) tetrakis(4-methylbenzenesulfonate) (4h).

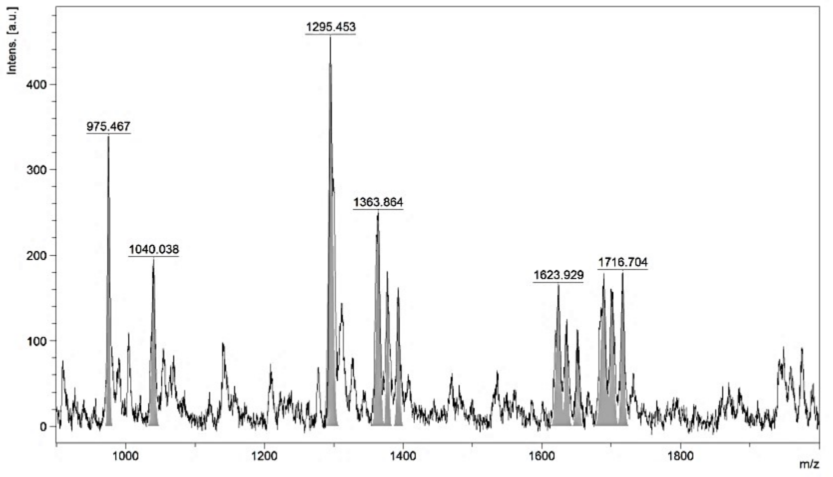

m/z	S/N	Quality Fac.	Res.	Intens.	Area
975.467	9		283	321	1807
1040.038	6		183	195	1350
1295.453	13		169	423	3427
1363.864	7		202	242	1994
1377.491	6		327	181	1263
1393.061	5		297	162	1114
1623.929	5		204	153	1362
1635.908	4		294	125	856
1651.971	4		299	113	674
1689.075	5		182	165	1646
1701.317	5		250	161	1262
1716.704	6		312	180	1186

Figure S33. HR-Mass spectra of porphyrin-5,10,15,20-tetrayltetrakis(benzene-4,1-diyl) tetrakis(4-methylbenzenesulfonate) (4h).
